# Dynamic Imine-Linked NIPU with Shape Memory, Self-Healing, and Prospects for Recyclability

**DOI:** 10.3390/polym18172081

**Published:** 2026-08-27

**Authors:** Kshitij S. Shinde, Muhammad Yasar Razzaq, Harald Rupp, Zviadi Katcharava, Wolfgang H. Binder, Anke Schadewald

**Affiliations:** 1 Institut für Kunststofftechnologie und Recycling e.V., Gewerbepark 3, 06369 Weißandt-Gölzau, Germany; harald.rupp@iktr-online.de (H.R.); anke.schadewald@iktr-online.de (A.S.); 2 Macromolecular Chemistry, Division of Technical and Macromolecular Chemistry, Faculty of Natural Sciences II (Chemistry, Physics, Mathematics), Institute of Chemistry, Martin Luther University Halle-Wittenberg, von-Danckelmann-Platz 4, 06120 Halle, Germanywolfgang.binder@chemie.uni-halle.de (W.H.B.); 3 Design of 3D-Printable Polymers Based on Regional Resources, Just Transition Center, Martin Luther University Halle-Wittenberg, 06099 Halle, Germany

**Keywords:** NIPU, imine, vitrimer, self-healing, shape memory effect, recyclability

## Abstract

Non-isocyanate polyurethanes (NIPUs) incorporating dynamic imine linkages were developed via UV-induced photopolymerization of methacrylated monomers. The system combines a non-isocyanate urethane (NIU) precursor derived from cyclic carbonate chemistry with an imine-containing (Vit) monomer obtained via Schiff base formation, enabling compositionally tunable networks with a high gel content (90–97 wt%) and thermal stability up to >190 °C. The increase in NIU content enhances network rigidity, varying the glass transition temperatures from 29.3 °C to 73.4 °C. The dynamic imine chemistry imparts outstanding multifunctionality, including efficient shape memory behavior, thermally triggered reprogrammability, and rapid surface self-healing behavior, even at a low content of imine-containing precursor (30 wt%). The optimized composition further provides a proof-of-concept of selective chemical depolymerization for monomer recovery. These results establish NIU-Vit networks as versatile, having recyclability prospects, and a multifunctional platform for sustainable polymers as replacements for the traditional isocyanate-based polyurethanes.

## 1. Introduction

A shape memory polymer (SMP) is a stimuli-responsive material capable of recovering its original permanent shape after being temporarily deformed. By utilizing a specific transition temperature (T_trans_), an SMP can be programmed into a temporary shape. This is achieved by heating the polymer above its T_trans_, deforming it into the desired configuration, and then cooling it below T_trans_ to fix the temporary form. Upon reheating the material above T_trans_, the polymer chains regain mobility, allowing them to relax and return to their thermodynamically favored conformation. The elastic strain energy stored during the initial deformation drives the contraction or relaxation of the molecular chains, resulting in the recovery of the polymer’s original shape [1,2,3].

Shape memory polyurethanes (SMPUs) are among the most extensively studied shape memory polymers and have demonstrated significant potential in applications ranging from stress sensors to actuators and intelligent textiles [4,5,6]. SMPUs can exist as both thermoplastic and thermoset systems. Thermoplastic SMPUs consist of phase-separated morphologies, in which the hard segments form reversible physical crosslinks through hydrogen bonding and microdomain aggregation, while the soft segments act as the switching phase responsible for the shape memory effect. Upon heating above the transition temperature (T_trans_), typically associated with the glass transition (T_g_) or melting temperature (T_m_) of the soft segments, chain mobility is restored, enabling deformation and subsequent fixation of a temporary shape upon cooling. In contrast, thermoset SMPUs contain permanent covalently crosslinked networks that define the permanent shape. During programming, elastic deformation is stored within the network, and upon reheating above T_trans_, this stored elastic energy is released, resulting in recovery of the original shape.

Thermoplastic SMPs are widely favored as they can be readily reshaped through melting or dissolution. However, thermoplastic systems are susceptible to creep under sustained load and compromised long-term mechanical integrity, particularly under elevated temperatures or prolonged stress conditions. Despite their excellent mechanical strength and chemical resistance, recycling remains a major challenge for crosslinked systems [7], which are often mechanically ground for reuse or chemically broken down into intermediates such as polyols or amines [8,9]. Traditional UV-cured polymers also suffer from this critical limitation: they are most often crosslinked thermosets, hence limiting the reshapability of the polymers once cured [10,11]. The concept of covalent adaptable networks (CANs) emerged from the need to develop a new class of materials that could possess thermoset-like properties while having the possibility of reconfiguration and rearrangement to achieve end-of-life reprocessability [12,13]. CANs, including vitrimers, are crosslinked networks possessing dynamic covalent bonds (DCBs) which can undergo reversible rearrangement on application of external stimuli such as heat, light or pH. Vitrimers are classified into associative CANs, wherein original dynamic bonds are broken and new ones reformed, either associatively or via dissociation. Therefore, the crosslink density of vitrimers remains almost constant, thus allowing the material to be reshaped without breaking the overall network and compromising its mechanical properties [14]. The commonly studied vitrimeric networks are based on transesterification [15], silyl ether exchange [16], vinylogous urethane [17], boronic esters [18], disulfides [19], and imine bonds [20,21].

The incorporation of dynamic imine linkages into polymer networks imparts vitrimer-like behavior, enabling crosslinked thermosets to be repaired and reshaped when subjected to elevated temperatures [22]. Primarily favored because of their catalyst-free and highly tunable nature, imine bonds (–C=NH or –C=NR), also known as reversible Schiff base linkages, are formed via condensation reactions between amines and aldehydes or ketones and exhibit dynamic covalent behavior through equilibrium-controlled reactions such as hydrolysis, transimination, and metathesis [23,24,25,26,27]. Among these, imine metathesis plays a crucial role in network rearrangement, allowing the simultaneous cleavage of existing bonds and formation of new ones while maintaining an overall constant crosslink density [28,29,30]. As a result, when the material is heated above its T_g_ and subjected to external stress, the network undergoes topological rearrangement via bond exchange, leading to stress relaxation and enabling permanent reshaping into a new configuration [31]. This dynamic adaptability endows imine-based vitrimeric polymers with a unique combination of properties, including malleability, shape memory behavior, self-healing capability, and enhanced recyclability [12,13,32].

Conventionally, polyurethanes are synthesized by reacting polyols with di- or polyisocyanates [33]. However, the use of isocyanates raises significant health and environmental concerns due to their toxicity and the use of hazardous reagents like phosgene in their production [7]. As a result, increasing research efforts are focused on developing non-isocyanate polyurethane (NIPU) routes, which aim to enhance sustainability, reduce toxic intermediates, and improve safety in line with green chemistry principles [34,35,36]. NIPUs exploit the ring-opening reaction of cyclic carbonates with (poly)amines, which can be further functionalized with photoreactive methacrylate groups to enable UV-curing [37,38,39]. Additionally, recent advances have focused on integrating shape memory functionality into NIPUs, enabling materials that can switch between a permanent shape and one or more temperature-programmable temporary shapes, governed by glass transitions or melting points, allowing materials to recover programmed shapes in response to external stimuli such as heat, light, or humidity [40,41,42,43].

In this work, a non-isocyanate polyurethane (NIPU) with vitrimer-like properties due to the presence of imine bonds is synthesized via UV-curing. One monomer is synthesized via Schiff base formation through the condensation of a methacrylate-containing aldehyde with a commercially available Jeffamine ED-900 (M-JED (Vit)). The second monomer (M-TOTDD), containing the non-isocyanate urethane (NIU) linkages, is obtained by reacting a diamine (4,7,10-Trioxa-1,13-tridecanediamine (TOTDD)) with cyclic carbonate, which was further followed by methacrylation. The NIU-Vit polymeric system is prepared with varying ratios of these two photoreactive monomers. The systems are initially evaluated with gel content determination, Fourier-transform infrared spectroscopy (FT-IR), thermogravimetric analysis (TGA), and dynamic mechanical analysis (DMA). On being determined as the most suitable formulation, NIU-Vit 70:30 is selected for further tests on tensile properties, shape memory effect and reprogrammability, and self-healing capabilities. Furthermore, a preliminary, exploratory assessment of selective chemical depolymerization is conducted to evaluate its feasibility, provide proof-of-concept insights, and qualitatively assess the obtained products. This class of materials, combining vitrimer-like behavior with NIPU chemistry, opens promising pathways for advanced, sustainable polymer applications.

## 2. Experimental Section

### 2.1. Materials

4-Hydroxybenzaldehyde (4-HBA, ≥98%, Carl Roth, Karlsruhe, Germany), methacrylic anhydride (MAAn, ≥94%, Sigma Aldrich, Burlington, MA, USA), 4,7,10-Trioxa-1,13-tridecanediamine (TOTDD, BASF, Ludwigshafen, Germany), ethylene carbonate (EC, 98%, Sigma Aldrich), hydroquinone (HQ, >99%, Carl Roth), triethylamine (TEA), ethyl phenyl(2,4,6-trimethylbenzoyl)phosphinate (TPO-L) SpeedCure photoinitiator was supplied by Arkema Sartomer (Colombes, France). 4-Dimethylaminopyridine (DMAP, 99%, Alfa Aesar, Ward Hill (Haverhill), MA, USA), Jeffamine ED-900 (Huntsman Holland BV, Botlek Rotterdam, The Netherlands), dichloromethane (DCM, Carl Roth), chloroform (Carl Roth), diethyl ether (Carl Roth), sodium hydroxide (Carl Roth), and sodium hydrogencarbonate (VEB Laborchemie Apolda, Apolda, Germany) were all purchased and used as received.

### 2.2. Methods

The curing of the resins was performed using an Anycubic cure 2 system (Shenzhen, China) with a UV-LED lamp (25 W) and a wavelength of 405 nm. The monomer mixtures were placed in Teflon molds and kept at a distance of 3–10 cm from the UV source. The exposure time was varied from 10 to 15 min. After curing, the samples were washed with isopropyl alcohol to remove any unreacted monomers from the surface.

Differential scanning calorimetry (DSC) measurements were performed using a DSC1 STAR System from Mettler Toledo (Greifensee, Switzerland) with heating and cooling rates of 10 and −10 K·min^−1^, respectively. The measurements were carried out in a flowing nitrogen atmosphere (30 mL·min^−1^) in the region −50 °C to 200 °C.

Thermogravimetric Analysis (TGA) was performed using a Mettler Toledo TGA2 (Greifensee, Switzerland). The heating rate was 5 K·min^−1^ with a 40 mL·min^−1^ flow of nitrogen. The samples were thermally degraded in a temperature range from 25 °C to 800 °C.

Fourier-transformed Infrared (FTIR) spectra were obtained from a Thermo Scientific Nicolet iS10 FTIR spectrometer (Waltham, MA, USA), equipped with a Golden Gate ATR unit. The measurement was conducted with 64 scans in the region of 400 to 4000 cm^−1^. The data analysis was conducted using OriginPro 2019 (Version 9.6.0.172).

Nuclear magnetic resonance (NMR) spectra were measured on an Agilent Technologies Varian Gemini 400 spectrometer (Santa Clara, CA, USA) at 27 °C with CDCl_3_ as solvent. For interpretation of the NMR spectra, the MestReNova software (version 9.0.1-13254) was utilized.

Dynamic mechanical analysis (DMA) in tensile mode was carried out on a Mettler Toledo Stare system DMA/SDTA861e (Greifensee, Switzerland) equipped with a 25 N load cell using photocured samples. The measurements were performed in temperature sweep mode from −50 to 200 °C with a constant heating rate of 1 K·min^−1^ in air, using an oscillation frequency of 10 Hz. During the measurements, a static strain of 1% and a dynamic strain of 0.25% were used. The glass transition (T_g_) was determined as the temperature at the maximum in the peak of the loss factor (tan*δ*) vs. temperature curve. The analysis was conducted using OriginPro 2019 (Version 9.6.0.172), and the curves were smoothed using the Loess method.

The tensile testing of the samples was carried out using a tensile tester (Zwick Z2.5, Ulm, Germany) with a standard dog-bone-shaped sample (similar to ISO 527-2:2025 [44]) cut from films. Samples have a total length of 75 mm (including the clamping area), gauge width of 5 mm, and a gauge length of 25 mm. The measurement was conducted at 23 °C and 50% r.h.

Microscopy was performed using an Olympus BH-2 reflected-light microscope (Olympus Corporation, Tokyo, Japan) equipped with a BH2-UMA illuminator. Images were acquired with an Olympus MTV-3 C-mount camera adapter coupled to an Olympus UC30 digital microscope camera using 2.5×, 10×, or 20× objective lenses, and analyzed using Olympus Stream Essentials 1.7 software.

### 2.3. Synthesis

#### 2.3.1. Synthesis of 4-Formylphenyl Methacrylate (4-FPMA)

The procedure was adopted from a previously published literature [31].

**Scheme 1:**
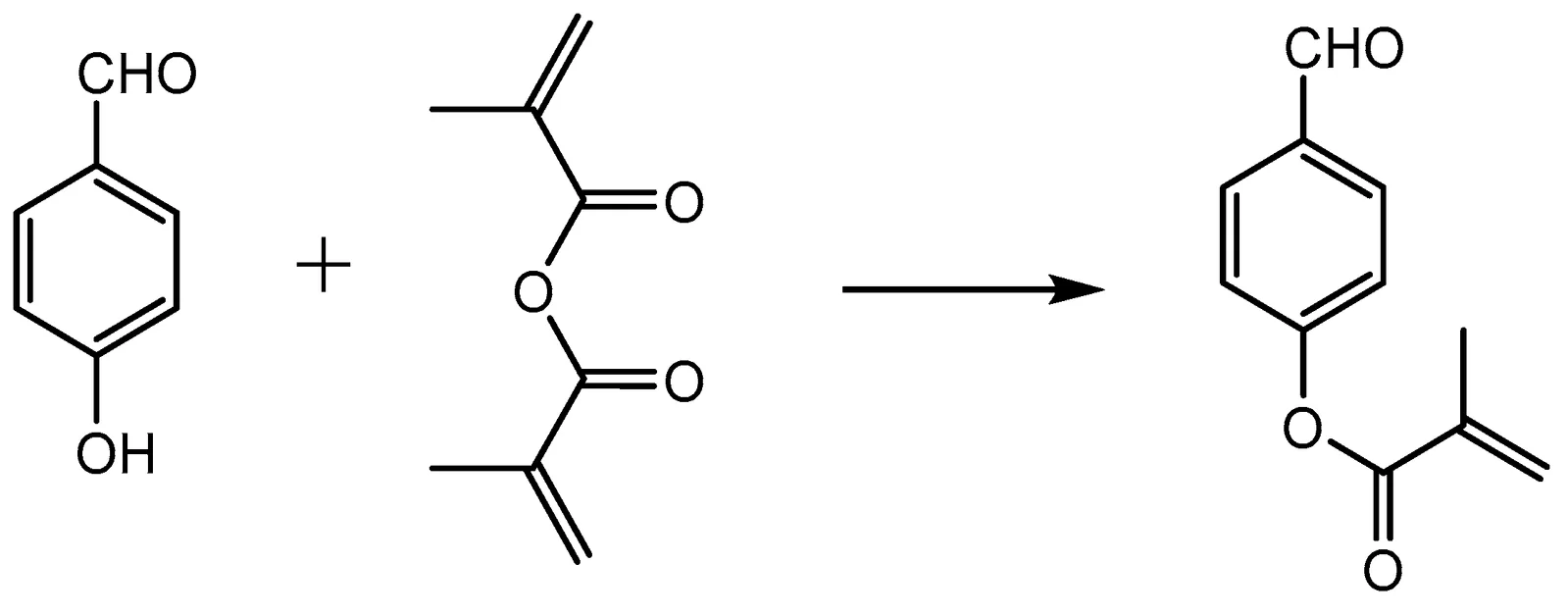
Synthesis of 4-FPMA.

In a three-necked flask (500 mL) under a nitrogen atmosphere, 4-hydroxybenzaldehyde (24.4 g, 0.20 mol) was dissolved in 200 mL of dichloromethane. Then, methacrylic anhydride (33.9 g, 0.22 mol, 33 mL) was added dropwise, followed by a small amount of 4-dimethylaminopyridine (DMAP) (0.5 g). The reaction mixture was magnetically stirred and refluxed at 40 °C for 14 h. Afterwards, the flask was removed from the bath and the product mixture was washed with 150 mL of saturated NaHCO_3_, 0.5 M NaOH, and water. The organic phase was separated and dried using anhydrous sodium sulfate. The brown DCM solution was passed through a layer of activated basic alumina (1 cm). Then, the solvent was removed under reduced pressure to obtain 28 g (yield 78%) of a viscous pale yellow liquid (see Scheme 1), which was directly used as obtained for the next synthesis step. For storage, a small amount of inhibitor 4-methoxyphenol MEHQ was added (100 ppm).

IR (ATR-IR): ν = 508 cm^−1^ (m), 625 cm^−1^ (m), 646 cm^−1^ (m), 709 cm^−1^ (w), 735 cm^−1^ (w), 786 cm^−1^ (w), 806 cm^−1^ (m), 856 cm^−1^ (m), 880 cm^−1^ (w), 944 cm^−1^ (m), 1012 cm^−1^ (m), 1111 cm^−1^ (s), 1156 cm^−1^ (m), 1206 cm^−1^ (s), 1291 cm^−1^ (m), 1319 cm^−1^ (m), 1387 cm^−1^ (w), 1453 cm^−1^ (w), 1503 cm^−1^ (w), 1598 cm^−1^ (m), 1637 cm^−1^ (w), 1695 cm^−1^ (m), 1735 cm^−1^ (m).

#### 2.3.2. Imination of 4-FPMA with JED (M-JED)

**Scheme 2:**
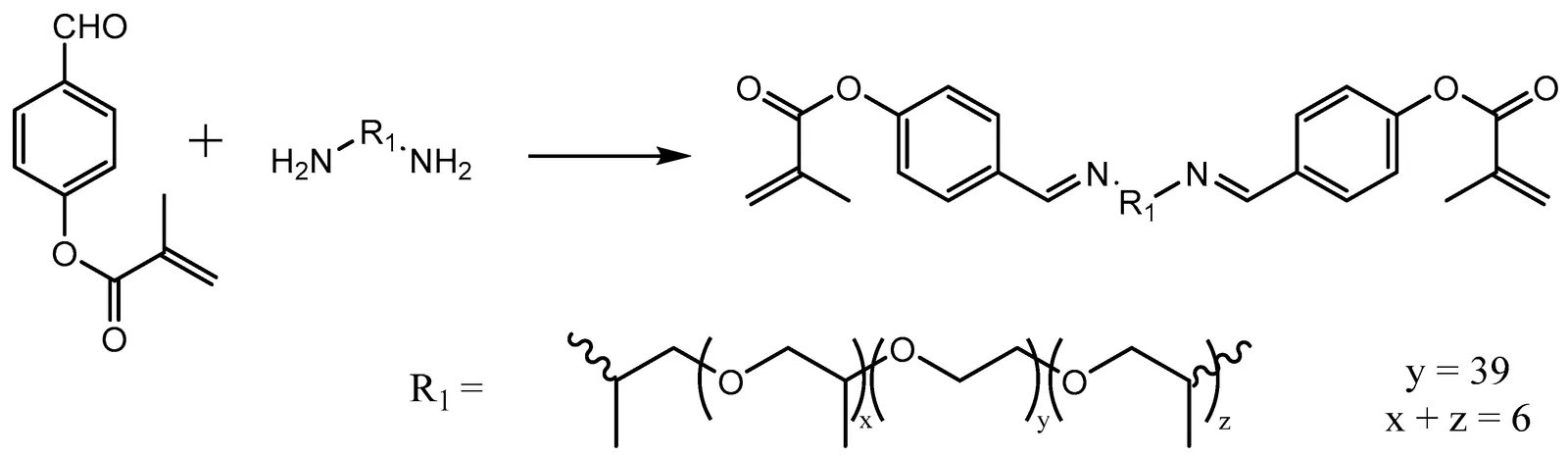
Synthesis of M-JED.

A mixture of 4-FPMA (3.8 g, 20 mmol), Jeffamine ED-900 (9.0 g, 10 mmol), and 5.7 g Na_2_SO_4_ was stirred in DCM (50 mL) at room temperature for 24 h. The reaction mixture was initially filtered to remove the Na_2_SO_4_ and then concentrated under reduced pressure and collected as a bright yellow viscous liquid (see Scheme 2).

^1^H-NMR (400 MHz, CDCl_3_) δ 8.27 (s, 2H), 7.72 (t, J = 10.3 Hz, 4H), 7.14 (d, J = 8.5 Hz, 4H), 6.34 (d, J = 12.3 Hz, 2H), 5.74 (m, 2H), 3.69–3.44 (m, 85H), 2.06–2.02 (m, 6H).

IR (ATR-IR): ν = 628 cm^−1^ (w), 815 cm^−1^, 881 cm^−1^ (w), 946 cm^−1^ (m), 1014 cm^−1^ (m), 1097 cm^−1^ (s), 1198 cm^−1^ (w), 1249 cm^−1^ (w), 1293 cm^−1^ (w), 1318 cm^−1^ (w), 1348 cm^−1^ (m), 1452 cm^−1^ (w), 1505 cm^−1^ (w), 1602 cm^−1^ (w), 1644 cm^−1^ (w), 1736 cm^−1^ (m), 2864 cm^−1^ (m).

#### 2.3.3. Synthesis of TOTDD-Diol

**Scheme 3:**
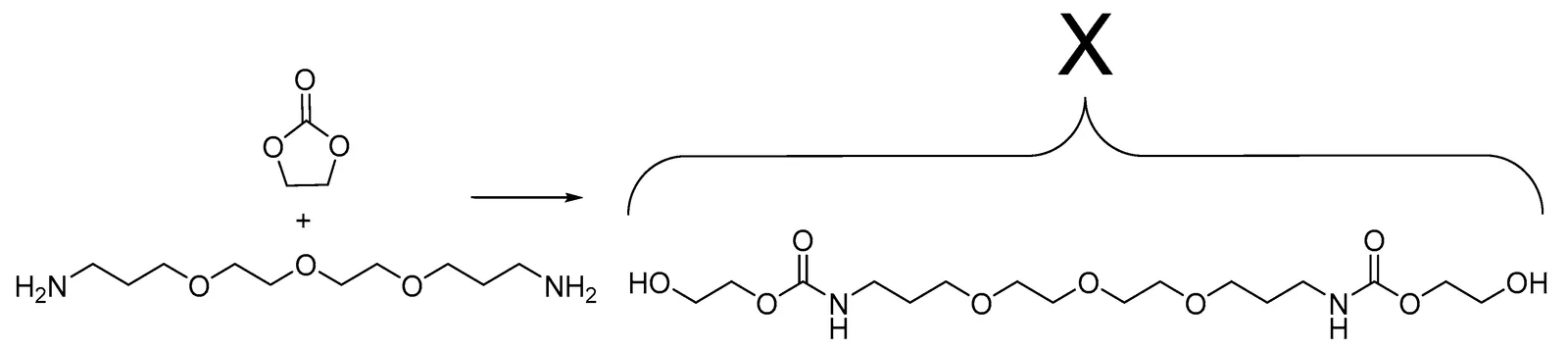
Synthesis of TOTDD-diol.

4,7,10-Trioxa-1,13-tridecanediamine (TOTDD) (10 g, 0.045 mol, 1 eq.), ethylene carbonate (EC) (8.8 g, 0.1 mol, 2.2 eq.), and CHCl_3_ (50 mL) were added in a round-bottomed flask and stirred at room temperature for 48 h. After removing CHCl_3_ in a rotatory evaporator, the obtained viscous liquid was precipitated in diethyl ether, washed with Et_2_O (100 mL) three times, and was dried in a vacuum oven at room temperature overnight. The product was a transparent viscous liquid (yield: 92%) (see Scheme 3).

^1^H-NMR (400 MHz, CDCl_3_) δ 5.80 (br, 2H), 4.15 (s, 4H), 3.75 (d, J = 3.9 Hz, 4H), 3.65–3.57 (m, 8H), 3.55 (t, J = 5.8 Hz, 4H), 3.27 (q, J = 6.1 Hz, 4H), 2.43 (s, 2H), 1.76 (p, J = 6.0 Hz, 4H).

IR (ATR-IR): ν = 3331 cm^−1^ (m), 2870 cm^−1^ (m), 1693 cm^−1^ (s), 1532 cm^−1^ (s), 1455 cm^−1^ (m), 1349 cm^−1^ (w), 1249 cm^−1^ (s), 1078 cm^−1^ (s), 1046 cm^−1^ (s), 885 cm^−1^ (m), 776 cm^−1^ (m), 502 cm^−1^ (s).

#### 2.3.4. Synthesis of M-TOTDD

**Scheme 4:**
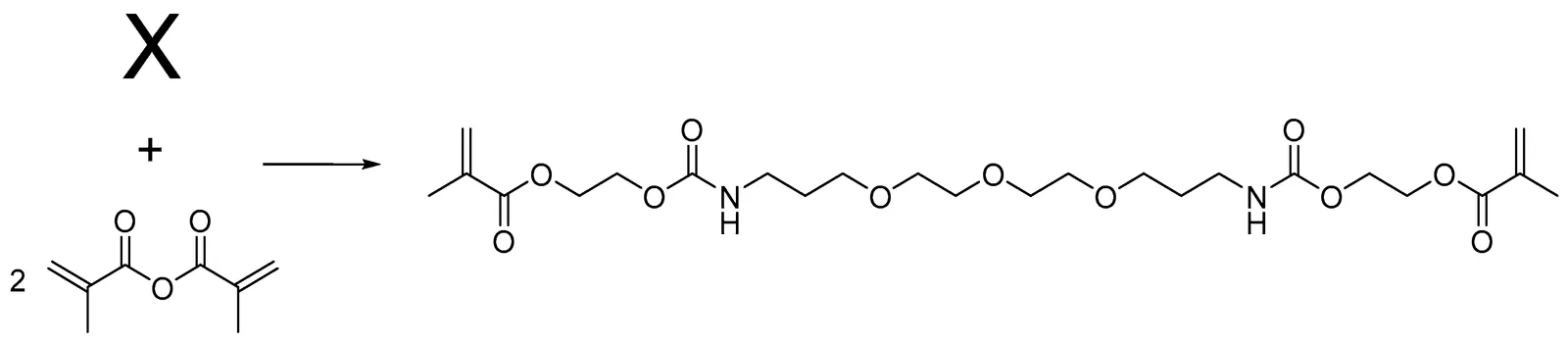
Synthesis of M-TOTDD.

TOTDD-diol (15 g, 0.036 mol, 1 eq.), DMAP (0.09 g, 0.0006 mol, 0.02 eq.), hydroquinone (HQ) (300 mg), and anhydrous CH_2_Cl_2_ (70 mL) were added in a round-bottomed flask in an ice bath under N_2_ atmosphere. Then, triethylamine (TEA) (14.04 mL, 10.2 g, 0.09 mol, 2.8 eq.) was dropwise added to the flask. Afterward, MAAn (13.2 g, 12.9 mL, 0.09 mol, 2.4 eq.) was dropwise added to the flask. The reaction mixture was stirred at room temperature for 24 h under N_2_ atmosphere. After that, saturated NaCl solution (200 mL) was added to get a two-phase separated mixture. The organic phase was collected and washed with HCl solution (1 M, 200 mL) three times, saturated NaHCO_3_ solution (200 mL) three times, and saturated NaCl solution (200 mL), and dried over anhydrous MgSO_4_. The CH_2_Cl_2_ was removed on a rotary evaporator at 20 °C, and the product was dried in a vacuum oven at room temperature overnight. The product was a yellow wax (yield: 68%) (see Scheme 4).

^1^H-NMR (400 MHz, CDCl_3_) δ 7.20–6.57 (m, 2H), 6.16–5.99 (m, 2H), 5.58 (t, J = 6.4 Hz, 2H), 4.52–4.04 (m, 8H), 3.70–3.45 (m, 11H), 3.36–3.11 (m, 4H), 2.13–1.89 (m, 6H), 1.85–1.62 (m, 4H).

IR (ATR-IR): ν = 3345 cm^−1^ (w), 2867 cm^−1^ (w), 1713 cm^−1^ (s), 1636 cm^−1^ (w), 1526 cm^−1^ (m), 1452 cm^−1^ (m), 1320 cm^−1^ (m), 1297 cm^−1^ (m), 1244 cm^−1^ (s), 1166 cm^−1^ (s), 1123 cm^−1^ (s), 943 cm^−1^ (m), 880 cm^−1^ (w), 814 cm^−1^ (w), 775 cm^−1^ (w), 734 cm^−1^ (w), 649 cm^−1^ (w), 594 cm^−1^ (w), 520 cm^−1^ (w).

#### 2.3.5. Synthesis of UV-Cured NIU-Vit Thermosets

The two monomers, M-TOTDD and M-JED, as well as the photoinitiator TPO-L, were directly poured into the molds. After mixing, the mixture was degassed in vacuum. The mold was then placed into the curing chamber for 10 min. The hardened samples were then removed from the mold, turned around, and placed in the curing chamber for another 5 min. The cured samples were then washed with isopropanol to remove any unreacted monomers on the surface and then dried. The samples were named NIU-Vit xNIU:xVit, where NIU and Vit are M-TOTDD and M-JED, respectively, and xNIU and xVit are their respective weight ratios. In Table 1, an example of one gram of UV-cured copolymer is shown to explain the weight ratios and the actual weight.

### 2.4. Determination of Gel Content

The gel content measurements of the cured samples were conducted with a solvent mixture of cyclohexane and DCM (70:30, volume%). The samples were dried in a vacuum oven at 50 °C for 24 h and then accurately weighed (*W*_0_). The samples were then submerged in a solvent at room temperature for 24 h. After removal from the solvent, the samples were dried initially in air at RT and then dried again at 50 °C for 24 h in a vacuum oven. The samples were weighed again accurately (*W*_1_). The gel content values were calculated according to Equation (1).

(1) $$Gel\;Content=\frac{W_{1}}{W_{0}}*100$$

As NIU-Vit 70:30 was chosen for shape memory tests, the gel content measurement of the cured sample was conducted additionally with DCM, THF, cyclohexane, and acetone.

### 2.5. Uniaxial Tensile Testing

The uniaxial tensile testing was conducted with a dog-bone shape (specimen type 1BA) with thicknesses ranging between 0.4 and 0.5 mm at a speed of 1 mm·min^−1^ for tensile modulus and 50 mm/min for tensile properties, in accordance with ISO 527-1:2019 [45]. Prior to the test, the samples were prepared as thin foils and cut into the dog-bone shape using the Coesfeld Materialtest (Hand-Kniehebelpresse, Dortmund, Germany) specimen cutter. The specimens were then conditioned in a climate-controlled room at 23 (±2) °C and 50 (±10) % r.h. for 72 h. The test was performed on five specimens of NIU-Vit 70:30 in the same climate conditions as those used for conditioning. The measurement results are provided in Tables S4 and S5.

### 2.6. Surface Self-Healing Assessment Test

To assess the self-healing capabilities, the samples of different weight ratios of NIU-Vit were assessed by gently cutting with a razor blade to produce “cracks”. After scratching, the samples were immediately viewed under optical microscopy as “before healing” samples at zeroth hour. The self-healing capabilities were then evaluated at room temperature (23 ± 2 °C und 50 ± 10% r.h) until sufficient healing was observed and by placing the samples in an oven at 100 °C (above the T_g_ of the cured polymers) for the duration of 2, 4, 6 and 12 h. The optical images were captured after each of the durations to observe the samples. For all the samples, the width of the scratch was measured at a minimum of three different points and then averaged. Changes in the crack widths before and after healing are reported in Tables S6 and S7. To maintain fairness, the same spot was captured under optical microscopy to measure the crack width.

### 2.7. Shape Memory Performance Test

The shape memory performance before and after permanent reprogramming was quantified using the bending test with a custom technique (Figure S22 and Table S8).

Flat specimens of NIU-Vit 70:30 (θ_0_ = 0 °C) before permanent programming were taken. The specimen was heated at 80 °C in an oven for 2 min, and then the specimen was bent into a U-shaped structure (θ*_u_* = 180 °C). The specimen was then kept in the U-shape for a further 10 min at 80 °C and then cooled down to RT. The specimen was then removed from the mold and immediately photographed (θ*_f_*).

Curved specimens of NIU-Vit 70:30 (θ_0_) after permanent reprogramming were taken. The specimen was heated at 80 °C in an oven for 5 min, and then the specimen was flattened using a 50 g weight (θ*_u_* = 180 °C). The flat specimen was kept for a further 10 min at 80 °C and then cooled down to RT. The weight was removed from the flat specimen and immediately photographed (θ*_f_*).

In both cases, the specimen was placed back into the oven at 80 °C for 120 s, and then the recovery angle was measured (θ*_r_*). Three specimens of NIU-Vit 70:30 were each tested in three cycles. The shape-fixity ratio (R_f_) and the shape-recovery ratio (R_r_) were calculated using Equations (2) and (3).

(2) $$Shape-fixity\;ratio=\frac{\theta_{f}}{\theta_{u}}*100$$

(3) $$Shape-recovery\;ratio=\frac{\theta_{u}-\theta_{r}}{\theta_{u}-\theta_{0}}*100$$

### 2.8. Chemical-Recycling (Selective Chemical Depolymerization) Test

The test was evaluated using two new NIU-Vit compositions. For the NIU-Vit 25:75 sample, the polymer was cut into small pieces and immersed in a 2:1 (*v*/*v*) THF:H_2_O (0.5 M H_2_SO_4_) solution. It was stirred for 48 h at 60 °C. Afterwards, the liquid phase was separated from the remaining solid fragments, neutralized, and extracted with DCM. The DCM phase was dried overnight under ambient conditions, followed by drying under vacuum at 50 °C. The recovered product (Extracted Compound **1** = EC1) was analyzed by FT-IR and NMR. The remaining solid fragments were also analyzed with FT-IR.

The second sample, NIU-Vit 75:25, was first subjected to the same acidic treatment. The residual solid obtained after acid treatment was subsequently treated with a 2:1 (*v*/*v*) MeOH:H_2_O (2 M NaOH) and stirred for 48 h at 60 °C to induce alcoholysis under basic conditions. After the reaction, the solvent mixture was separated from the remaining solid fragments. The mixture was extracted with DCM at neutral pH (6–7, Extracted Compound **2** (EC2) and acidic pH (1–2, Extracted Compound **3** (EC3)) and dried (overnight under ambient conditions, followed by drying under vacuum at 50 °C) separately. The recovered extracts were analyzed using FT-IR and NMR.

## 3. Results and Discussion

### 3.1. Synthesis of UV-Cured NIPU

**Figure 1:**
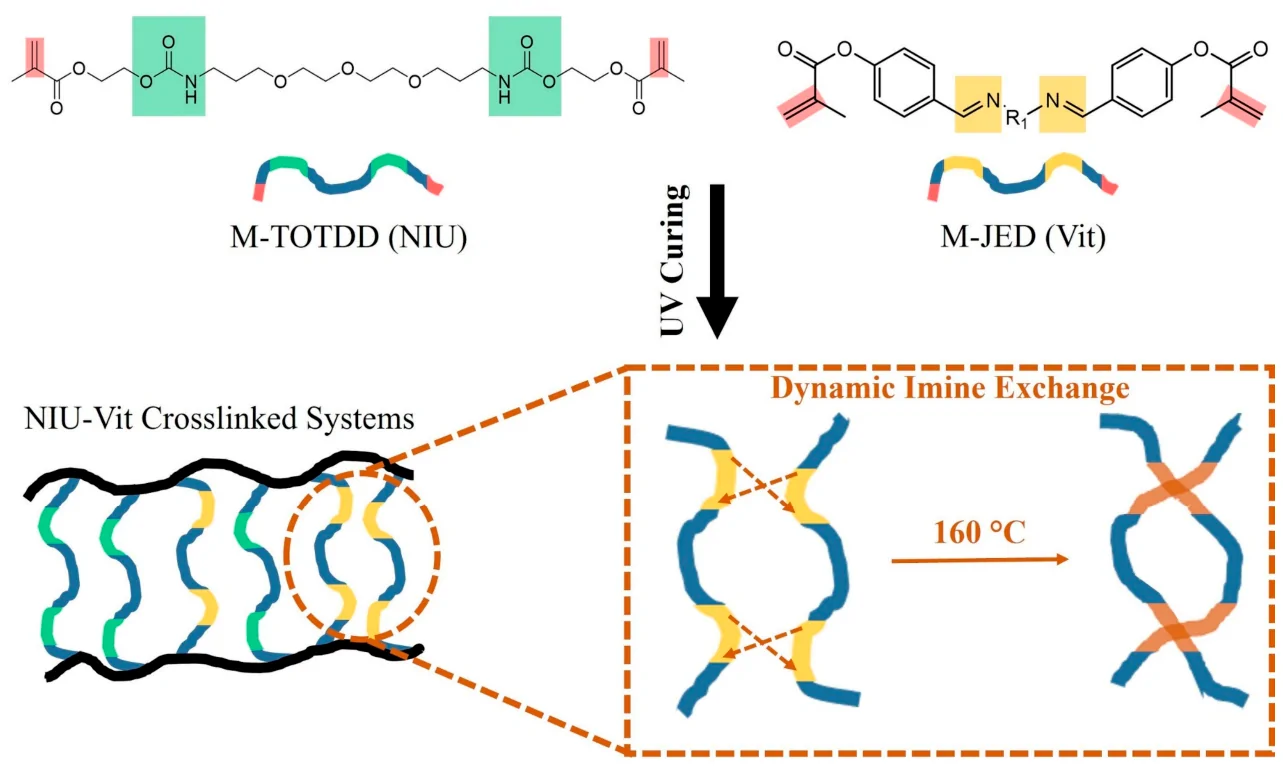
A schematic representation of the photopolymerization of the NIU-Vit network systems.

The chemical structures of the precursors used for the synthesis of the NIPU with dynamic networks are shown in Figure 1. In the first step, 4-FPMA was synthesized by reacting 4-HBA with MAAn (Scheme 1). The aldehyde group was then further reacted with the primary amines of Jeffamine ED-900 (diamine, JED, M_n_ = 900 g·mol^−1^) to form modified JED (M-JED) with the vitrimer-like (Vit) imine (C=N) linkages via a Schiff base (nucleophilic addition–elimination) mechanism, releasing water as a by-product [46]. The ED series of Jeffamine consists of poly(ethylene glycol)-based polyether diamines, which impart water solubility to its products. The modified JED (M-JED) is a bis-imine monomer containing two aromatic methacrylate moieties, connected by an R_1_ spacer (defined in Scheme 2) through two imine bonds, as confirmed by IR and ^1^H NMR spectroscopy (Figures S2 and S3).

The second reaction sequence involves the stepwise preparation of the non-isocyanate-urethane-linked dimethacrylate precursor using TOTDD. In the first step, the diamine reacts with ethylene carbonate (EC) to produce an intermediate with non-isocyanate urethane linkages (NIU) and terminal hydroxyl groups (X = dihydroxyurethane, Scheme 3). In the second step, the carbamate linkage-containing intermediate was further reacted with MAAn to yield a urethane-linked dimethacrylate product (M-TOTDD, Scheme 4), as confirmed by IR and ^1^H-NMR spectroscopy (Figures S7 and S8).

The introduction of polymerizable methacrylate groups at chain ends of both monomers makes them suitable as reactive monomers or crosslinkers for UV-curable resins. Subsequent photopolymerization of methacrylated M-TOTDD and M-JED at varying weight ratios (Table 2), using ethyl phenyl(2,4,6-trimethylbenzoyl)phosphinate (TPO-L) as the photoinitiator, produced crosslinked NIPU networks with tunable properties and functionalities (detailed procedure in Section 2.3.5). TPO-L, a phosphine oxide-based photoinitiator, enables fast curing in UV-curable systems, providing high reactivity and excellent depth of cure [31]. The samples were named NIU-Vit xNIU:xVit, where NIU and Vit correspond to M-TOTDD and M-JED, respectively, and xNIU and xVit represent their respective weight ratios.

**Figure 2:**
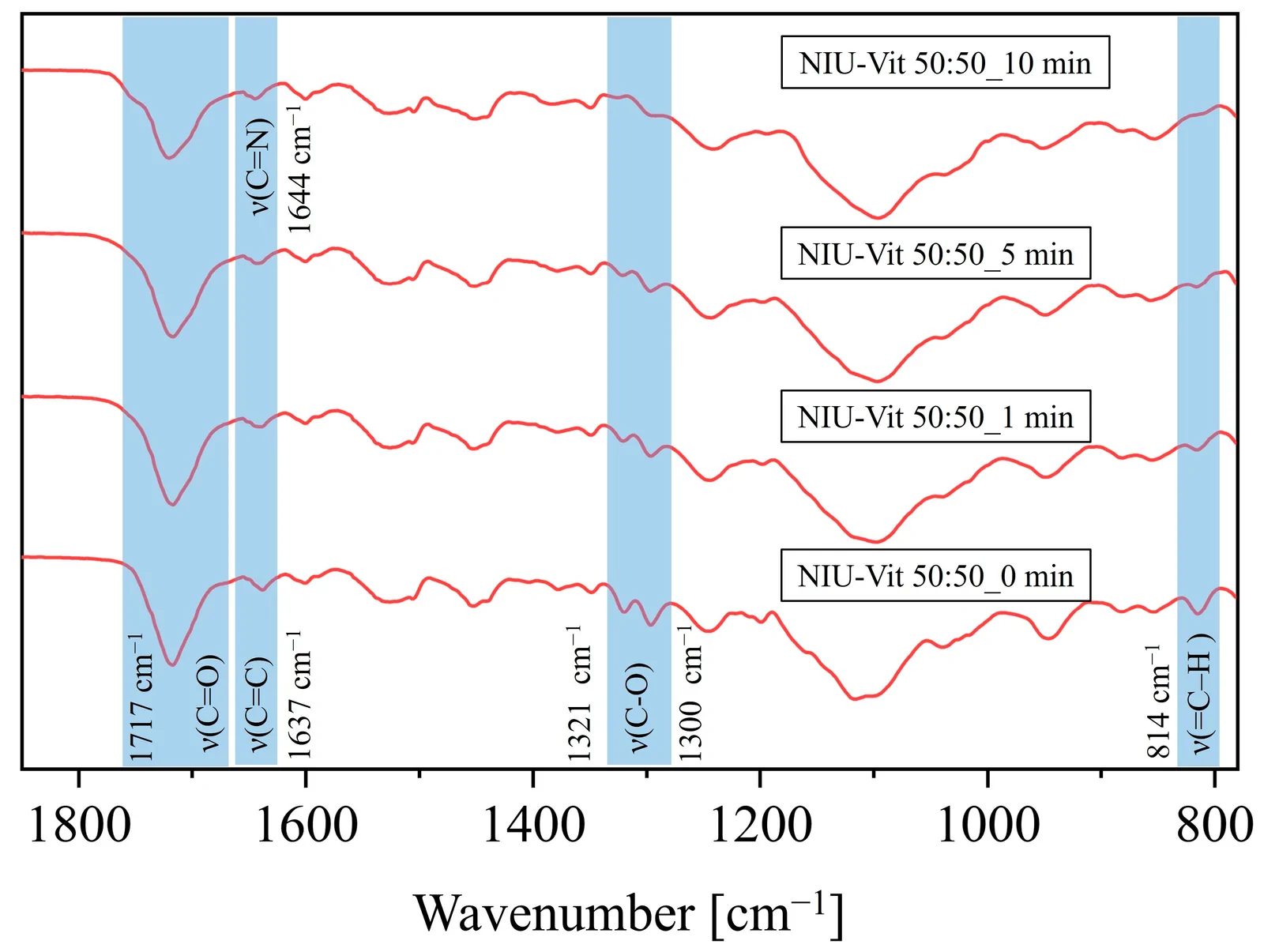
The effect of UV exposure time on photopolymerization curing in NIU-Vit 50:50.

The progress of the photopolymerization of the monomers, specifically the methacrylate groups, was monitored using FT-IR spectroscopy using NIU-Vit 50:50 as a representative system. The effect of UV exposure time on curing, proceeding via free-radical polymerization of the C=C double bonds, was evaluated through the characteristic changes associated with consumption of the vinyl double bond and changes in the ester environment (Figure 2 and Figure S10). Initially, an overlap of the bands of imine [ν(C=N)] and vinyl ν(C=C) resulted in a peak at 1637 cm^−1^. As polymerization progressed and the C=C bonds were consumed, this band shifted to 1644 cm^−1^, corresponding predominantly to the imine ν(C=N) vibration. This is further supported by the complete disappearance of the band at 814 cm^−1^, corresponding to the =C–H deformation of the vinyl group [47]. In the fingerprint region, the loss of peaks at 1300 and 1321 cm^−1^ is consistent with the disappearance of the ν(C–O) doublet (1300–1320 cm^−1^) reported for methacrylate curing [48]. Additionally, minor shifts in the carbonyl region (from 1717 to 1720 cm^−1^) were observed due to altered mobility and local environments following network formation [49].

The FT-IR spectra of the copolymers with varying weight ratios are shown in Figure S11. With an increase in the NIU weight ratio (0 → 100), the intensity of the band at 1246 cm^−1^ (eventually shifting to 1242 cm^−1^), attributed to ester C–O–C asymmetric stretching, increased due to the higher functionality per mass of M-TOTDD. Concurrently, the band at ~1600 cm^−1^, associated with aromatic C=C stretching (from M-JED), decreased. A reduction in the imine ν(C=N) band at 1644 cm^−1^ was also observed with decreasing M-JED content. In the carbonyl region, a pronounced shift from ~1750 to 1704 cm^−1^ occurred, particularly between NIU-Vit 0:100 and 30:70, indicating the increasing contribution of urethane carbonyl groups and their involvement in hydrogen bonding. This is further supported by an increase in the ~3341 cm^−1^ broad peak, associated with the hydrogen-bonded N-H stretching from urethane.

The extent of network formation following photopolymerization was evaluated by determining the gel content (G) of the copolymers (see Tables S1 and S2 for more details). The cured samples were extracted using a solvent mixture of cyclohexane and DCM (70:30 *v*/*v*%), showing an increasing trend in gel content from NIU-Vit 30:70 (G = 90 wt%) to NIU-Vit 100:0 (G = 97 wt%). Such a solvent mixture was chosen because pure DCM by itself induced swelling-related cracks in the cured samples. Additionally, NIU-Vit 70:30 was evaluated using DCM, THF, cyclohexane, and acetone. In all cases, the gel content remained in the range of 93–99 wt%, confirming the robustness of the network structure.

### 3.2. Determination of Thermal and Mechanical Properties

**Figure 3:**
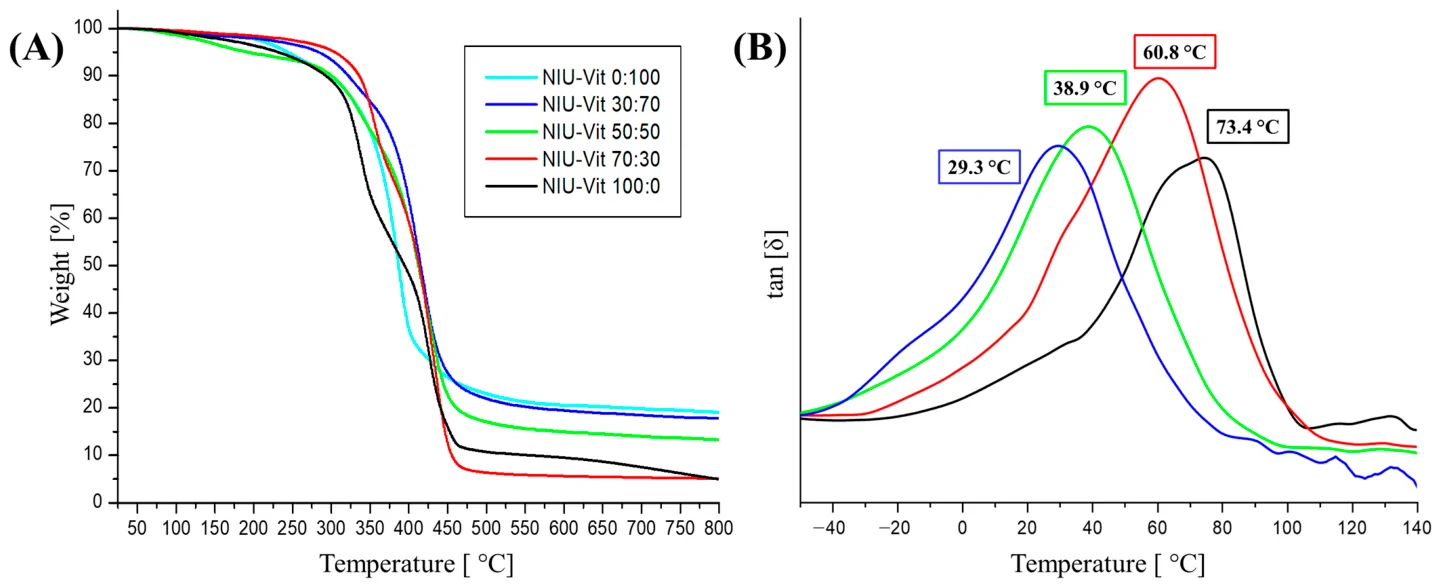
(**A**) The TGA curves for different NIU-Vit systems and (**B**) measured tan(δ) values for different NIU-Vit systems.

The thermal stability of the copolymer series was investigated with TGA under an inert atmosphere (see Figure 3A and Table S3). Up to 150 °C, the NIU-Vit 50:50 sample showed the highest weight loss of 3.3 wt%. This was attributed to the loss of moisture, consistent with the presence of hydrophilic polyether (PEG-like) segments. The minimum thermal stability of all samples was at least 191 °C, as indicated by T_5%_ (temperature at 5 wt% weight loss). The photocured NIU-Vit 100:0 exhibited a distinct two-step thermal degradation (Figure S12). The first step, with a weight loss of around ~40% between 230 and 377 °C, was attributed to the degradation of the relatively thermally labile urethane linkages, leading to the release of CO_2_ and amines [50]. The second step, with a weight loss of around ~45% between 377 and 520 °C, was attributed to the degradation of the crosslinked polymeric backbone [37]. The maximum degradation temperatures (T_d,max_) for these steps were 338 °C and 425 °C, respectively. On the other hand, NIU-Vit 0:100 underwent a relatively single-step degradation between 160 and 700 °C with the T_d,max_ at 387 °C and an overall weight loss of 79%. As previously seen in the literature with similar systems [31], the composition with the highest M-JED (NIU:Vit 0:100) showed the highest residual char mass of 19.1% at 800 °C. This could be attributed to the higher carbonization resulting from the deterioration of aromatic and aliphatic groups of M-JED. A decreasing trend in residual char content was observed with increasing NIU fraction (Table S3).

The thermal and thermomechanical properties of the networks were investigated using differential scanning calorimetry (DSC) and dynamic mechanical analysis (DMA), respectively. As seen in Figure S13, DSC measurements for the crosslinked copolymers did not reveal a discernible T_g_ transition with a heating rate of 10 K·min^−1^. This behavior is typical of highly crosslinked thermosets, where the glass transition is associated with only a small change in heat capacity (ΔCp) due to restricted polymer chain mobility, with T_g_ increase generally correlating to increased crosslink density [51]. Additionally, the presence of strong urethane hydrogen bonding further restricts chain segmental mobility [52]. As DMA is more sensitive to the mechanical relaxations associated with T_g_, it was carried out at varied temperatures [53]. It revealed a clear composition-dependent increase in T_g_ for the UV-cured NIU-Vit networks (Figure 3B). The T_g_ increased from 29.3 °C (30:70) to 38.9 °C (50:50), 60.8 °C (70:30), and 73.4 °C (100:0), while the 0:100 composition was too soft for shape memory investigations, indicating a T_g_ below ambient temperature. The monotonic increase in the T_g_ with an increase in NIU weight fraction reflects the enhanced rigidity of the crosslinked network due to higher crosslink density, arising from the incorporation of shorter and more rigid M-TOTDD segments. Furthermore, hydrogen bonding associated with NIU contributes additional restrictions to segmental motion, further elevating the T_g_. The presence of a single, albeit broad, T_g_ across all compositions suggests good network homogeneity. The T_g_ of 60.8 °C of NIU-Vit 70:30 provided a sufficiently elevated transition temperature for the intended shape memory investigations compared to NIU-Vit 30:70 and 50:50, whose T_g_ values were closer to ambient temperature. At the same time, NIU-Vit 70:30 contains imine groups relevant to dynamic network behavior, unlike NIU-Vit 100:0. Additionally, the higher T_g_ values provide a higher usable service temperature. Therefore, NIU-Vit 70:30 was selected for further investigations.

The mechanical response of NIU-Vit 70:30 under ambient conditions was evaluated by uniaxial tensile testing at room temperature. The detailed conditions of the test are provided in Section 2.5. The test revealed a tensile modulus (E) of 74.4 ± 9.1 MPa, indicating a relatively soft crosslinked polymer. The stress at maximum load (σ_m_) and at break (σ_b_) were 10.3 ± 1.2 and 10.2 ± 1.3 MPa, respectively, while the corresponding strains (ε_m_ and ε_b_) were 18.04 ± 2.38% and 18.06 ± 2.36%, respectively. The negligible differences between stress and strain at maximum load and at break indicate that the material fails almost immediately after reaching peak stress, with minimal post-yield deformation. Overall, these results suggest that the polymer is a soft and moderately flexible material with modest tensile strength. The stress–strain curves are provided in Figure S14.

### 3.3. Investigations of Shape Memory Effect, Reprogrammability and Self-Healing Capabilities

**Figure 4:**
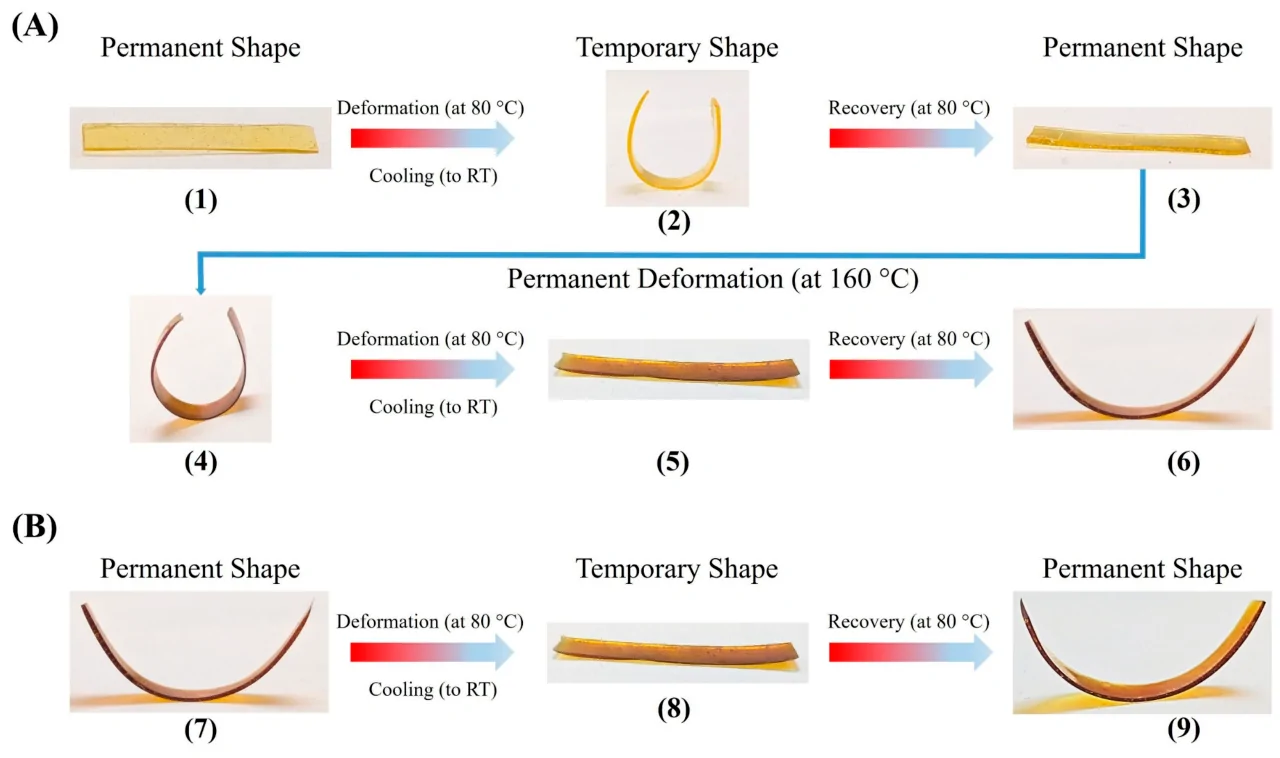
(**A**) Shape memory effect and reprogrammability assessment of NIU-Vit 70:30 as well as (**B**) shape recovery test for the newly tailored permanent shape.

In Figure 4A, the SMP capabilities of NIU-Vit 70:30 are displayed. The thin foils (~0.5 mm) were heated to 80 °C, well above the T_g_ of the network measured with DMA (60.8 °C), deformed into a curved shape, and cooled to room temperature to fix the deformed temporary shape (Figure 4(A1,2)). Upon reheating above the T_g_, the temporary curved shape was lost to quickly recover the original permanent shape (Figure 4(A3), Video S1). In the next step, permanent reprogramming via imine metathesis was then demonstrated. The NIU-Vit 70:30 foil was initially temporarily deformed at 80 °C, fixed to a rod, and then subsequently heated at 160 °C for 1.5 h under constraint. These conditions were selected based on preliminary screening of different reprogramming temperatures and time durations, as they provided pronounced reprogrammability among the conditions investigated. At this temperature, imine exchange reactions enabled topological rearrangement, resulting in a new permanent shape (Figure 4(A4)). To verify reprogrammability, the foil was reheated to 80 °C, straightened into a new temporary shape, and cooled (Figure 4(A5)). Upon reheating above T_g_, the foil recovered toward the newly programmed permanent shape, although not completely. The specimen approached but did not fully reach the programmed permanent shape, indicating partial shape recovery (Figure 4(A6), Video S2). However, this “partially recovered” configuration effectively becomes the new permanent shape, as subsequent cycles of deformation and recovery do not visually degrade the recovery level (Figure 4(B7–9)). The incomplete recovery may arise from insufficient bond exchange or incomplete network rearrangement due to limited time or temperature [54,55]. The shape memory performance before and after permanent reprogramming was quantified using the bending test with a custom technique (see Section 2.6 and Table S8). The shape-fixity ratio (R_f_) and the shape-recovery ratio (R_r_) of the NIU-Vit 70:30 were calculated as 93.3 ± 1.3% and 98.4 ± 0.9%, respectively, before permanent reprogramming (Figure 4(A1–3)), and 97.8 ± 0.6% and 89.1 ± 3.3%, respectively, after permanent reprogramming (Figure 4(B7–9)). The optimization of the reprogramming conditions will be the focus of future work to achieve full recovery.

As self-healing capabilities become increasingly important for extending service life by improving durability and reliability, combining different self-healing mechanisms is essential for materials such as NIPUs to replace traditional polyurethanes across their diverse range of applications [56,57]. To assess the surface self-healing behavior of NIU-Vit 70:30 (along with the controls NIU-Vit 100:0 and NIU-Vit 0:100), samples were gently cut with a razor blade to generate surface “cracks.” The materials were then either stored at RT (23 °C and 50% r.h.) or at 100 °C. The samples were examined at different times, beginning at 0 h (“Before Healing”), using optical microscopy (detailed procedure in Section 2.6).

The self-healing capability of materials relying solely on hydrogen bonding is immensely low [58]. This was demonstrated by NIU-Vit 100:0, which showed negligible reduction in crack width even after 12 h at 100 °C (Figure S15A, Table S7). Alternatively, the presence of imine bonds encourages self-healing at milder conditions via dynamic exchange reactions [27], as observed in NIU-Vit 0:100, which exhibited partial crack closure at RT after 40 h and substantial closure within 2 h at 100 °C (Figure S15B, Tables S6 and S7).

**Figure 5:**
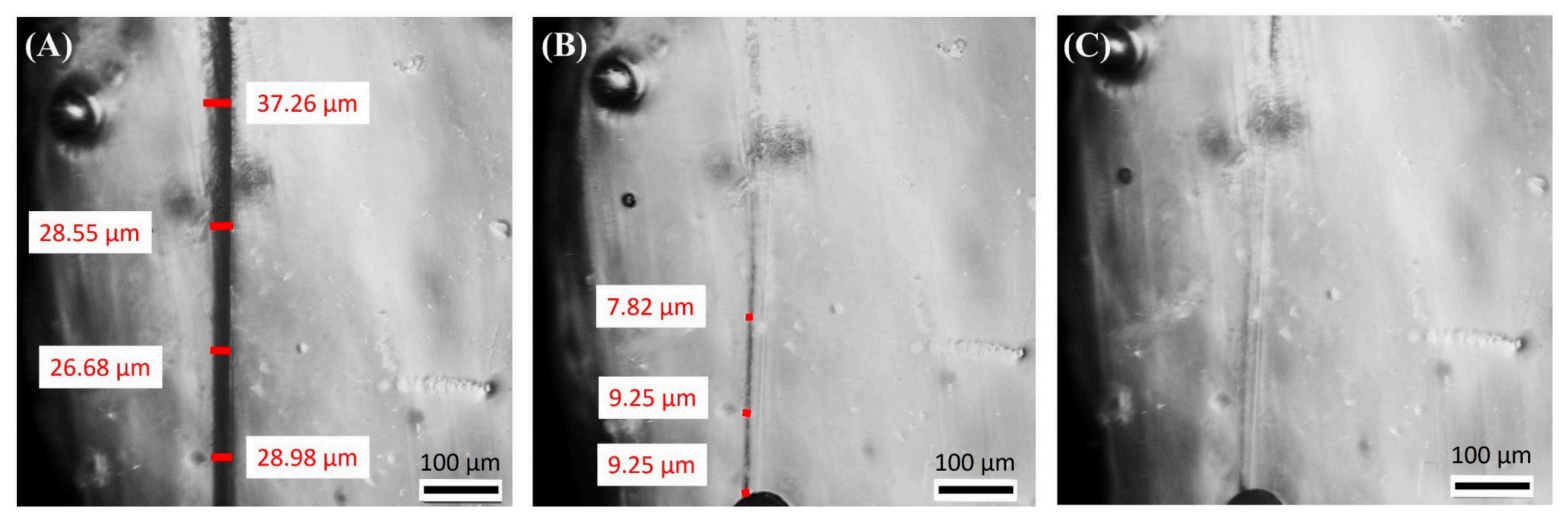
The assessment of the surface self-healing behavior of NIU-Vit 70:30 at room temperature (23 °C and 50% r.h.) at (**A**) 0 h “before healing,” (**B**) after 24 h and (**C**) after 40 h.

**Figure 6:**
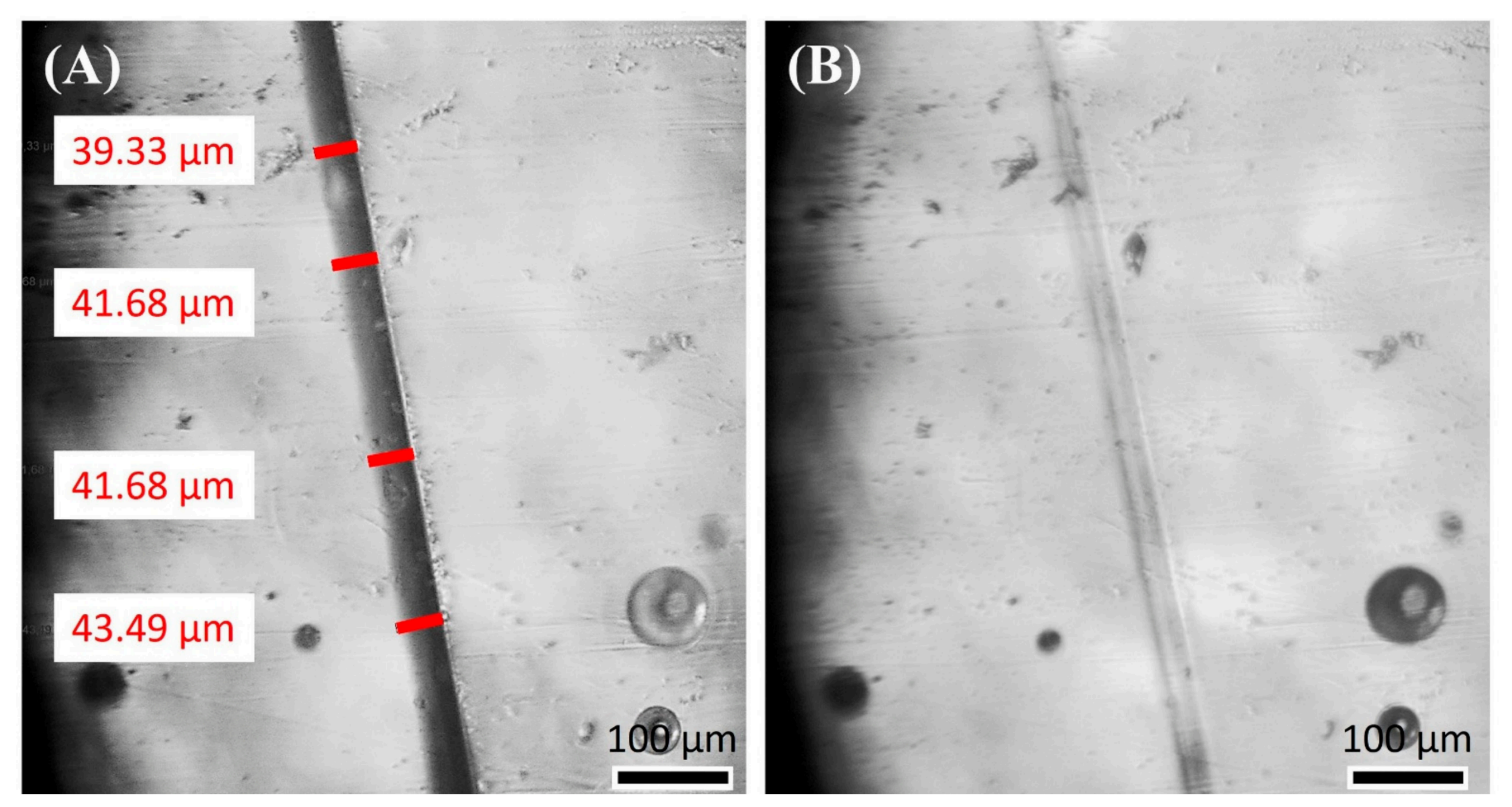
The assessment of the surface self-healing behavior of NIU-Vit 70:30 at 100 °C at (**A**) 0 h “Before Healing,” and (**B**) after 2 h.

The incorporation of hydrogen bonding and imine functionalities into NIPUs, like in PUs, should generate synergistic effects that facilitate self-healing in rigid crosslinked polymers at reduced temperatures or over shorter timescales [59]. Consistent with this expectation, NIU-Vit 70:30 displayed pronounced surface crack closure at RT, reducing the crack width by 71% within 24 h and achieving complete closure within 40 h, leaving only a faint scar (Figure 5, Table S6). This is not unusual, as multiple studies have reported such a synergistic behavior at RT with PUs [60,61,62]. This healing process of NIU-Vit 70:30 can be further accelerated by heating the sample at 100 °C. The visible crack was completely closed within 2 h, again leaving only a visible scar at the former cut site (Figure 6, Table S7).

### 3.4. Prospects for Recyclability of the NIU-Vit Systems

A preliminary qualitative chemical-recycling study to evaluate its feasibility and provide a proof-of-concept for selective chemical depolymerization was undertaken. The study was carried out using two new compositions of NIU-Vit systems (see Experimental Section 2.8), namely NIU-Vit 25:75 and 75:25. These samples were selected to remain close to the two extremes of the previously tested NIU-Vit series (NIU-Vit 30:70 and 70:30), while slightly increasing the proportion of the major monomer in each composition. This approach made it possible to focus on the recovery of each individual monomer at a higher proportion, while still preserving the influence of the other component.

**Figure 7:**
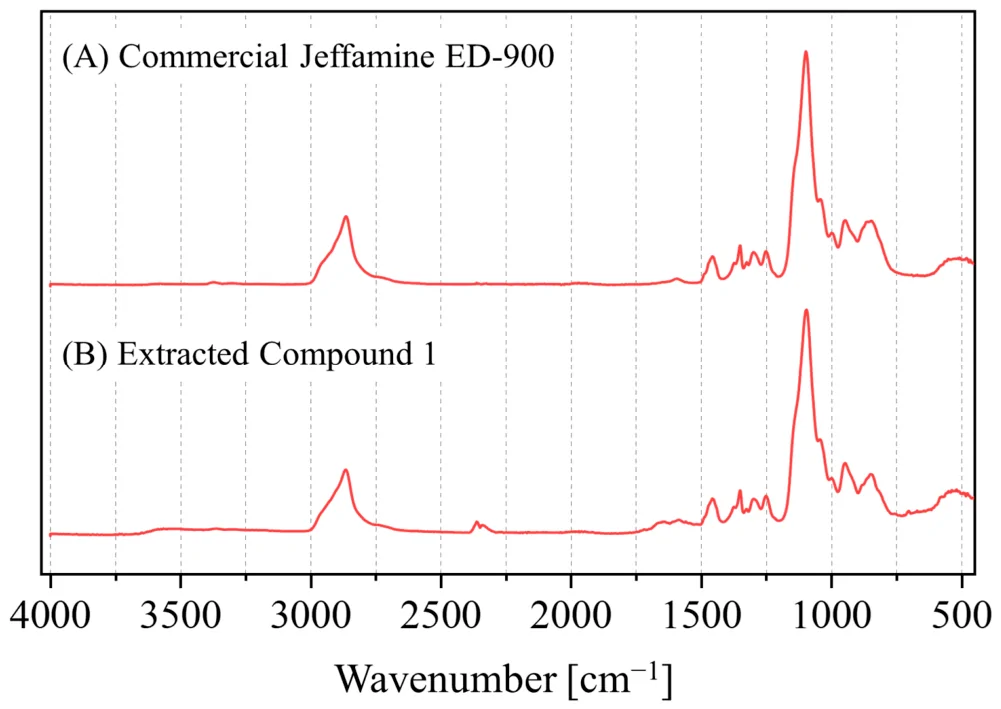
FT-IR comparison between (**A**) commercial Jeffamine ED-900 and (**B**) Extracted Compound **1** (EC1).

**Scheme 5:**
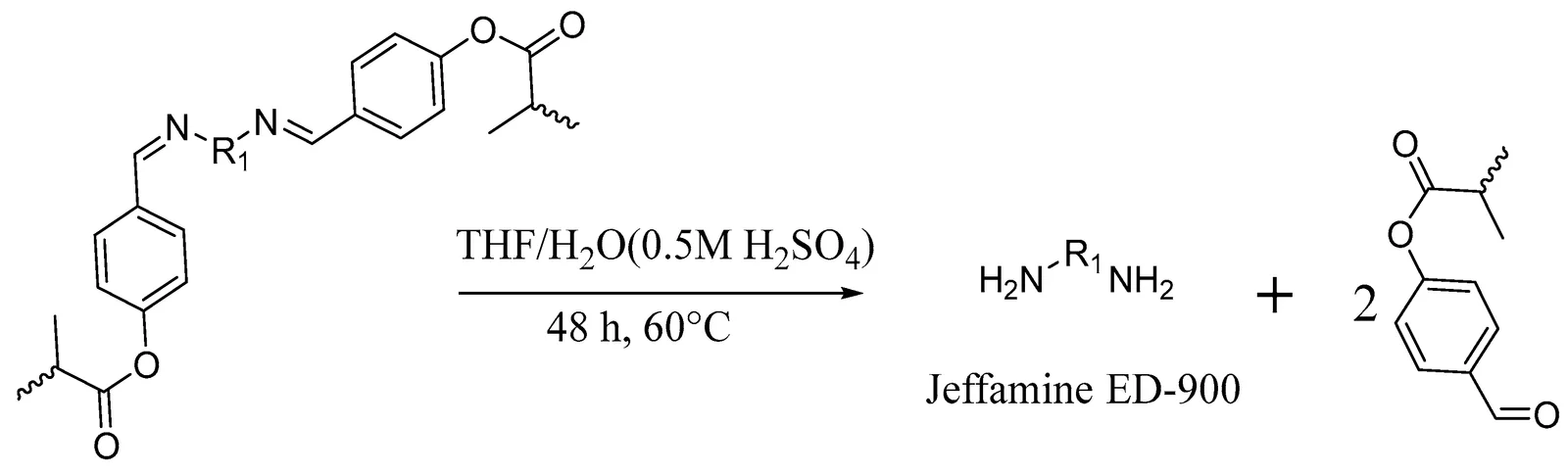
Acid-catalyzed hydrolysis of imines in NIU-Vit systems to retrieve Jeffamine ED-900.

In the first experiment, the sample NIU-Vit 25:75 was treated with a 2:1 (*v*/*v*) THF:H_2_O (0.5 M H_2_SO_4_) solution, where it was stirred for 48 h at 60 °C. The recovered product (EC1) and the remaining solid fragments (SF1) were analyzed. The acid-catalyzed hydrolysis of imines is well known, with the imine bond reverting back to the precursor amine and carbonyl-containing compound [27,63]. The acidic water protonates the imine nitrogen to form an iminium ion, increasing the electrophilicity of the C=N bond. Nucleophilic attack by water forms a carbinolamine intermediate, which undergoes proton transfers and C–N bond cleavage, releasing the amine and regenerating the carbonyl compound [64]. This was reflected in the FT-IR of the residual solid fragments of the NIU-Vit 25:75 (SF1), which showed disappearance of the imine peak at 1645 cm^−1^ and a shift in the peak from 1720 cm^−1^ (carbonyl-ester) to 1699 cm^−1^ (see Figure S17). Furthermore, the FT-IR and NMR analyses of EC1 (Figure 7 and Figure S16) matched commercial Jeffamine ED-900, confirming the recovery of the starting material. The possible reaction is shown in Scheme 5.

Under the same acidic conditions, the residual solid fragments (SF2) of the second sample, NIU-Vit 75:25, also showed changes similar to NIU-Vit 25:75. The small imine shoulder at 1645 cm^−1^ disappeared while a shift in the peak from 1715 cm^−1^ (carbonyl-ester) to 1701 cm^−1^ (see Figure S18) was observed. Continuing the selective chemical depolymerization process, the acid-treated residual solid fragments were treated with 2:1 (*v*/*v*) MeOH:H_2_O (2 M NaOH) to induce alcoholysis under basic conditions at 60 °C for 48 h. Because pH strongly influences ionization, solubility, and extractability of organic compounds [65,66], the mixture was extracted with DCM at neutral pH (6–7, EC2) and acidic pH (1–2, EC3), and analyzed after drying.

**Figure 8:**
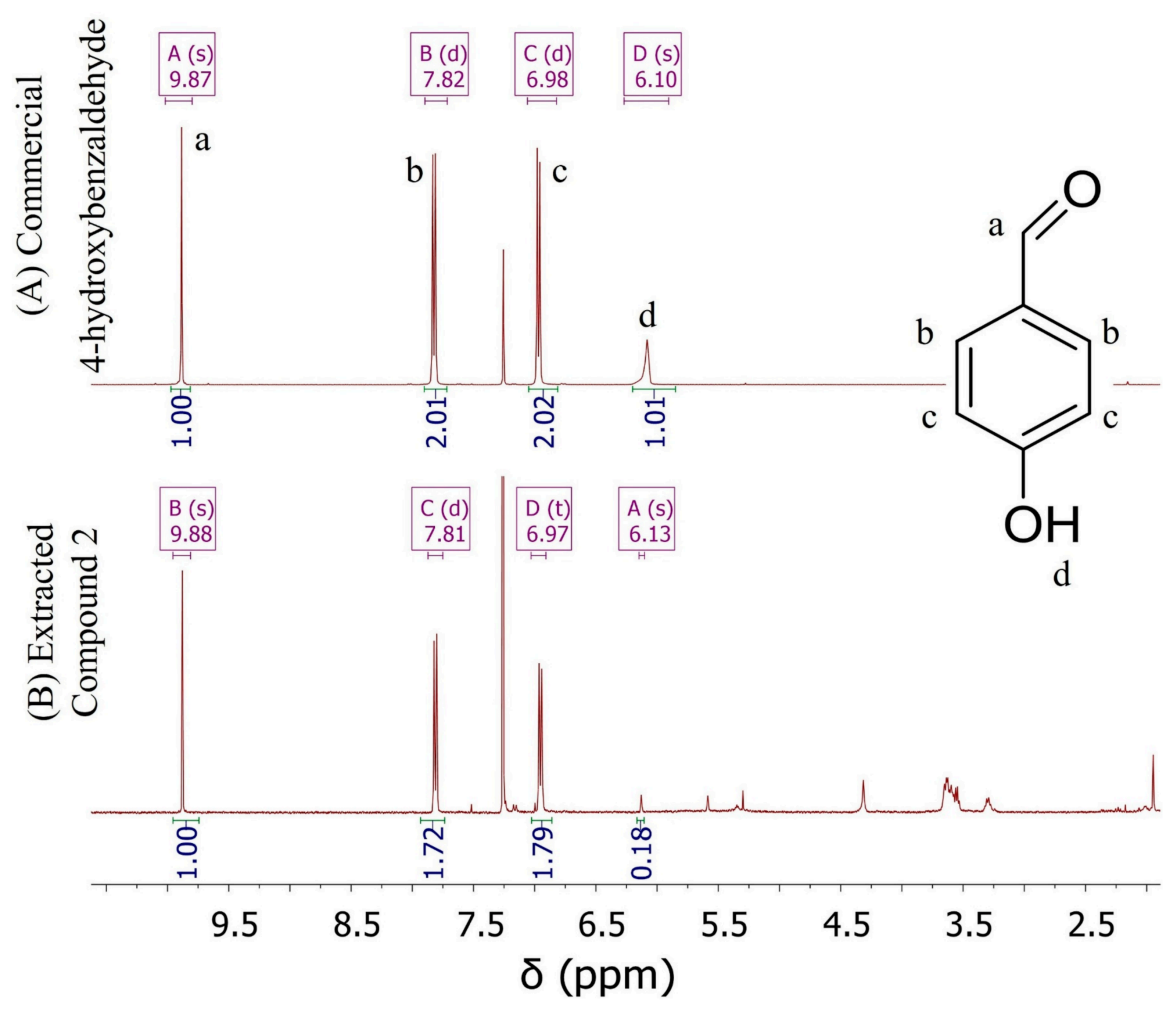
The NMR comparison of (**A**) commercial 4-hydroxybenzaldehyde and (**B**) Extracted Compound **2** (EC2).

**Scheme 6:**
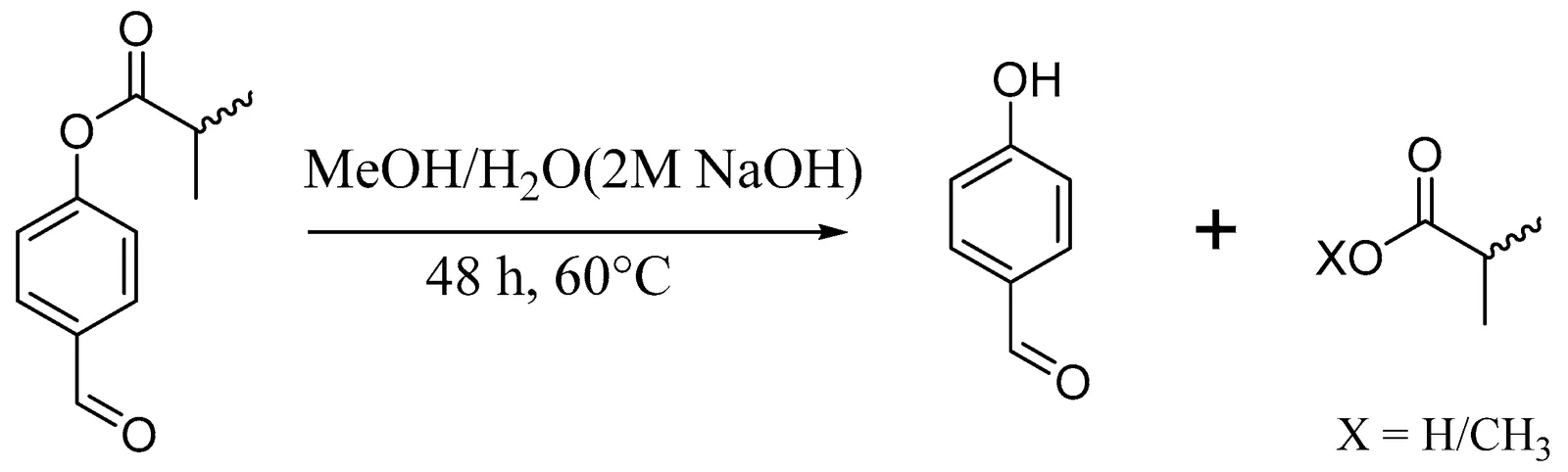
Basic alcoholysis in NIU-Vit systems leading to recovery of 4-HBA.

**Scheme 7:**
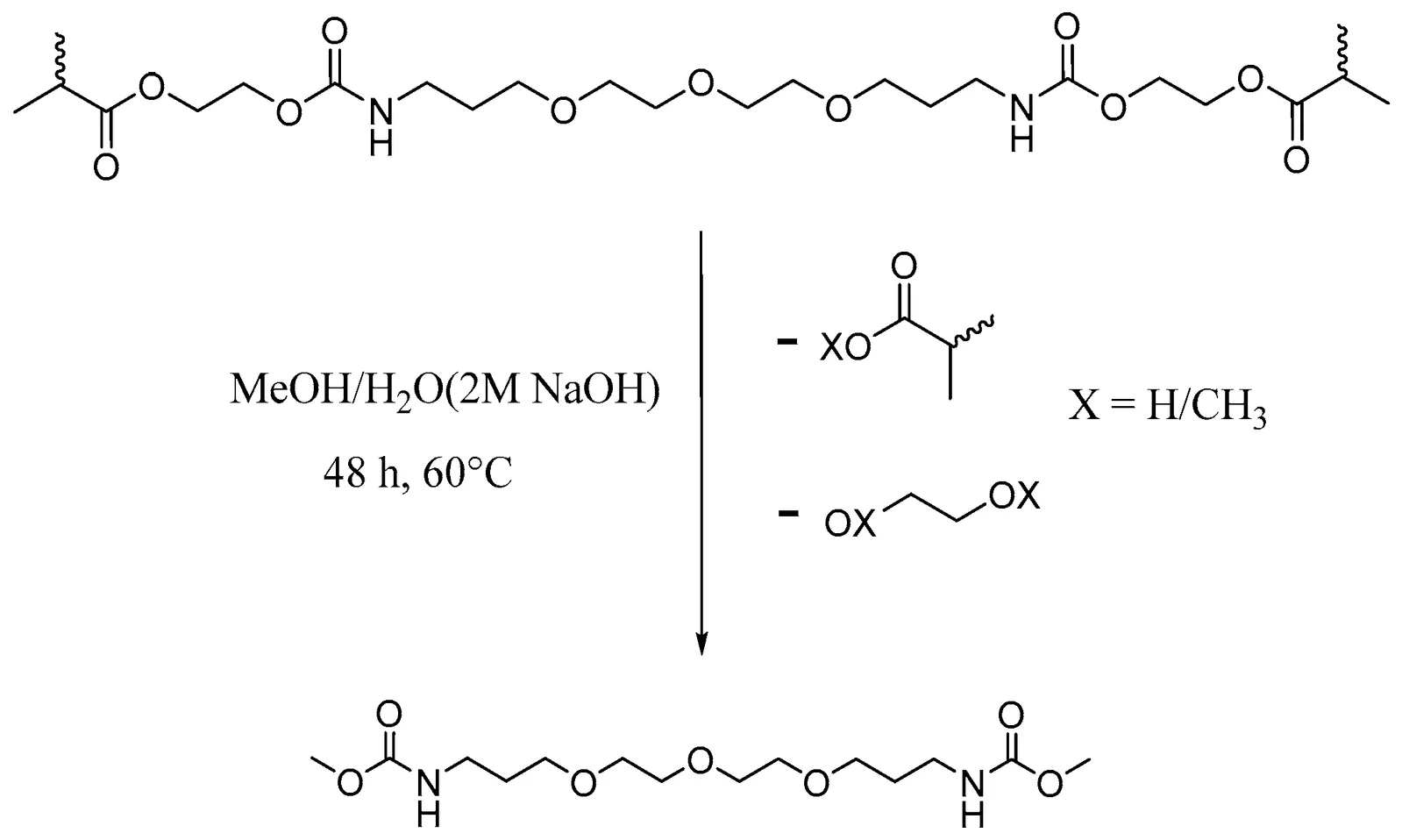
Basic alcoholysis in NIU-Vit systems leading to recovery of bis(methyl carbamate) of TOTDD.

The basic alcoholysis of carbonyl-containing groups is well studied [67]. The recovered EC2 at neutral pH matched with commercial 4-HBA in FT-IR and NMR. Comparison with the in-house spectral library indicated an 85.0% match to 4 HBA (Figure S19). Although the NMR spectrum contained some minor impurities, the signals belonging to 4-HBA dominated overall (Figure 8). On the other hand, the extract obtained at acidic pH after basic alcoholysis, EC3, showed a mixture of 4-HBA and a bis(methyl carbamate) of TOTDD (see Figure S20). The proposed reaction pathway is shown in Scheme 6 and Scheme 7.

**Figure 9:**
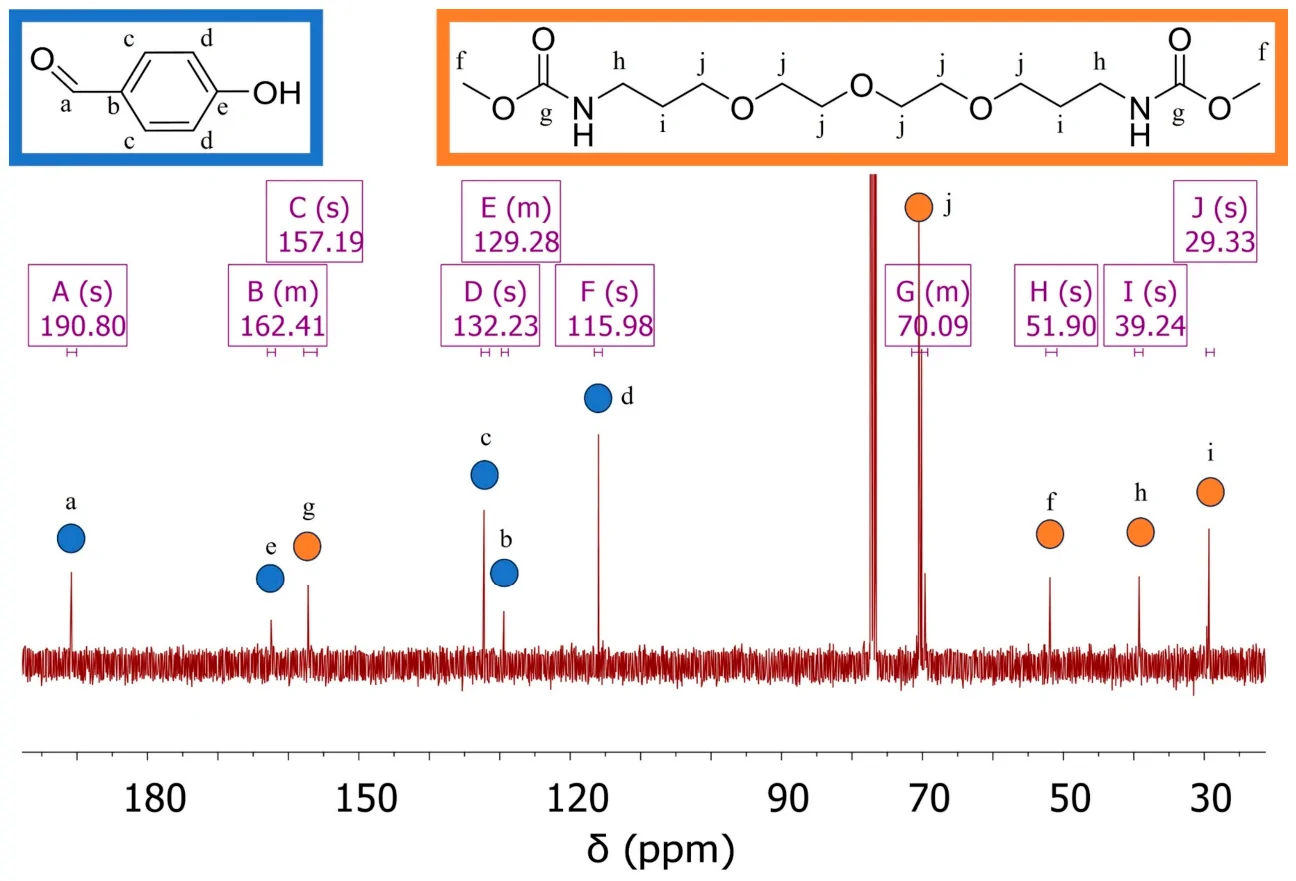
^13^C-NMR of the Extracted Compound **3** (EC3) at acidic pH after basic alcoholysis.

The signals at 9.84, 7.77 and 6.99 ppm, albeit slightly shifted, correspond to 4-HBA, while those at 5.31, 3.72–3.54, 3.29 and 1.77 ppm belong to the bis(methyl carbamate) of TOTDD (see Figure S20). The signals at 4.15 and 3.77 ppm, belonging to –CH_2_– groups of the reacted ethyl carbonate, were absent. However, the 3.72–3.54 ppm region integrated to 18 protons instead of the expected 12 protons. This suggests the possibility of an overlap of the –O–**CH_3_** groups of the ester with the –CH_2_– groups of TOTDD (Figure S21). To confirm this, ^13^C-NMR was also performed. The peak at 51.90 ppm confirms the formation of methoxy groups (–O–**CH_3_**) (Figure 9). The recovered bis(methyl carbamate) of TOTDD can be used as a precursor for transurethanization, another pathway for NIPU synthesis [68,69,70,71]. Therefore, the undertaken preliminary recycling study has successfully established a proof-of-concept for selective chemical depolymerization, providing a foundation for further optimization and refinement of the recovery process of the starting materials.

## 4. Conclusions

In this work, a series of non-isocyanate polyurethane (NIPU)-based crosslinked copolymer networks containing dynamic imine linkages were successfully synthesized via UV-induced photopolymerization of methacrylated NIU (consisting of non-isocyanate urethane linkages) and Vit (consisting of imine linkages) monomers.

FT-IR confirmed the efficiency of photopolymerization and network formation by providing verification of the consumption of the methacrylate C=C bonds of the NIU-Vit series consisting of varied ratios of the photoreactive monomers. This was further supported by high gel content values (90–97 wt%) across the compositions, indicating a high degree of crosslinking.

The NIU-Vit networks exhibited good thermal stability (T_d_ > 190 °C), with degradation behavior governed by composition. The Vit-rich compositions showed a high residual char of 19.1% at 800 °C due to higher carbonization from deterioration of aromatic and aliphatic groups of the Vit component.

The DMA showed a systematic increase in T_g_ from 29.3 °C to 73.4 °C with the increase in the rigid NIU component, reflecting enhanced crosslinking density and reduced chain mobility. Based on its T_g_ (60.8 °C), the NIU-Vit 70:30 composition was selected for further evaluation, as higher T_g_ values provided a sufficiently elevated transition temperature for the intended shape memory investigations.

The NIU-Vit 70:30 composition exhibited moderate tensile modulus (74.4 ± 9.1 MPa) and flexibility. The composition demonstrated excellent shape memory effect at a programming temperature of 80 °C. By exploiting thermally activated imine exchange at 160 °C, permanent reprogrammability of the composition was also demonstrated. The composition further exhibited efficient surface self-healing, achieving complete crack repair within 40 h at room temperature or 2 h at 100 °C due to the synergistic dual presence of hydrogen bonds and dynamic imine linkages.

A preliminary qualitative chemical-recycling study to evaluate its feasibility and provide a proof-of-concept for selective chemical depolymerization was undertaken. It was demonstrated that the networks are capable of selective depolymerization under acidic and basic conditions to recover key monomers like Jeffamine ED-900, 4-hydroxybenzaldehyde, and a bis(methyl carbamate) derivative of 4,7,10-trioxa-1,13-tridecanediamine (TOTDD), which can serve as a precursor for NIPU synthesis via transurethanization.

Overall, the NIU-Vit networks are versatile multifunctional systems that combine good thermal and moderate mechanical performance with shape memory and reprogrammability, surface self-healing capabilities, and a promising prospect for recyclability. These features highlight their potential as sustainable alternatives to conventional isocyanate-based polyurethanes for advanced functional applications.

## Figures and Tables

**Table 1:** Weights and weight ratios of the different NIU-Vit systems.

Entry No.	Sample Name	M-TOTDD (NIU)		M-JED (Vit)	
		Weight (g)	Weight Ratio (xNIU)	Weight (g)	Weight Ratio (xVit)
1	NIU-Vit 100:0	1.0	100	0.0	0
2	NIU-Vit 70:30	0.7	70	0.3	30
3	NIU-Vit 50:50	0.5	50	0.5	50
4	NIU-Vit 30:70	0.3	30	0.7	70
5	NIU-Vit 0:100	0.0	0	1.0	100

**Table 2:** Composition of the weight ratios (xNIU:xVit) of different NIU-Vit systems.

Entry No.	Sample Name	M-TOTDD (NIU)	M-JED (Vit)
		Weight Ratio (xNIU)	Weight Ratio (xVit)
1	NIU-Vit 100:0	100	0
2	NIU-Vit 70:30	70	30
3	NIU-Vit 50:50	50	50
4	NIU-Vit 30:70	30	70
5	NIU-Vit 0:100	0	100

## Data Availability

The original contributions presented in this study are included in the article/Supplementary Material. Further inquiries can be directed to the corresponding author.

## Supplementary Materials

The following supporting information can be downloaded at: https://www.mdpi.com/article/10.3390/polym18172081/s1, Figure S1: FT-IR spectrum of 4-FPMA; Figure S2: ^1^H-NMR spectrum of M-JED; Figure S3: FT-IR spectrum of M-JED; Figure S4: TGA thermogram of M-JED; Figure S5: ^1^H-NMR spectrum of TOTDD-diol; Figure S6: FT-IR spectrum of TOTDD-diol; Figure S7: ^1^H-NMR spectrum of M-TOTDD; Figure S8: FT-IR spectrum of M-TOTDD; Figure S9: TGA thermogram of M-TOTDD; Figure S10: Full spectra of the effect of UV exposure time on photopolymerisation curing in NIU-Vit 50:50; Figure S11: FT-IR spectra of the NIU-Vit networks with varying compositions; Figure S12: The DTG curve of NIU-Vit 100:0 and 0:100; Figure S13: DSC thermograms of different NIU-Vit systems; Figure S14: The stress-strain curves of the uniaxial tensile testing of five specimens of NIU-Vit 70:30; Figure S15: Control samples for surface self-healing behavior consisting of before and after microscopy images of (A) NIU-Vit 100:0 and (B) NIU-Vit 0:100; Figure S16: The NMR comparison of (A) commercial Jeffamine ED-900 and (B) Extracted Compound **1** (EC1); Figure S17: FT-IR comparison of NIU-Vit 25:75 before (in black) and after (in red, SF1) acidic hydrolysis; Figure S18: FT-IR comparison of NIU-Vit 75:25 before (in black) and after (in red, SF2) acidic hydrolysis; Figure S19: FT-IR comparison between (A) Extracted Compound **2** (EC2) at neutral pH and (B) commercial 4-HBA as reference; Figure S20: ^1^H-NMR of the Extracted Compound **3** (EC3) at acidic pH after basic alcoholysis; Figure S21: ^1^H-NMR of the extracted of bis(methyl carbamate) of TOTDD after basic alcoholysis; Figure S22: The bending test with a custom technique was used to quantify the shape memory performance of NIU-Vit 70:30 (A) before and (B) after permanent reprogramming; Table S1: Gel content of different NIU-Vit systems with cyclohexane:DCM 70:30 (v/v%); Table S2: Gel content of NIU-Vit 70:30 in different solvents; Table S3: Characteristic degradation temperatures at 5, 50, 80 and 90 wt% loss, as well as the char residue of different NIU-Vit systems; Table S4: Results of tensile modulus after uniaxial tensile testing; Table S5: Results of different tensile properties after uniaxial tensile testing; Table S6: Results of surface self-healing behavior of NIU-Vit 70:30 with controls of NIU-Vit 0:100 and 100:0 at room temperature (23 ± 2 °C und 50 ± 10% r.h); Table S7: Results of surface self-healing behavior of NIU-Vit 70:30 with controls of NIU-Vit 0:100 and 100:0 at 100 °C; Table S8: The parameters and results for the bending test with a custom technique to quantify the shape memory performance of NIU-Vit 70:30 before and after permanent reprogramming; Video S1: Shape recovery to the original permanent shape (flat) from a temporary curved shape (Figure 4(A2,3)). Video speed has been increased by ~5×; Video S2: Shape recovery to the original permanent shape (curve) from a temporary flat shape (Figure 4(A5,6)). Video speed has been increased by ~4×.
