# Supplementary material for: Dynamic Imine-Linked NIPU with Shape Memory, Self-Healing, and Prospects for Recyclability
Source: Polymers (Basel). 2026 Aug 27;18(17):2081. doi: 10.3390/polym18172081 (PMC13568353; doi:10.3390/polym18172081)
Supplement: Supplementary file 1 [file polymers-18-02081-s001.zip › polymers-4461012-Supplementary Information-ok.pdf]

SUPPLEMENTARY INFORMATION

# Dynamic Imine-Linked NIPU with Shape Memory, Self-Healing, and Prospects for Recyclability

*Kshitij S. Shinde*[*a], Muhammad Y. Razzaq*[* a], Harald Rupp*[a], *Zviadi Katcharava*[b,c], *Wolfgang H. Binder*[b], *Anke Schadewald*[a]

[a] Institut für Kunststofftechnologie und -recycling e.V., Gewerbepark 3, 06369 Weißandt-Gölzau, Germany

[b] Macromolecular Chemistry, Division of Technical and Macromolecular Chemistry, Faculty of Natural Sciences II (Chemistry, Physics, Mathematics), Institute of Chemistry, Martin Luther University Halle-Wittenberg, von-Danckelmann-Platz 4, D-06120 Halle, Germany.

[c] Design of 3D-Printable Polymers Based on Regional Resources, Just Transition Center, Martin-Luther-University of Halle-Wittenberg University, 06099 Halle, Germany.

E-mail: kshitij.shinde@iktr-online.de, yasar.razzaq@iktr-online.de, harald.rupp@iktr-online.de, zviadi.katcharava@chemie.uni-halle.de, wolfgang.binder@chemie.uni-halle.de, anke.schadewald@iktr-online.de

1. Synthesis of 4-formylphenyl methacrylate (4-FPMA)

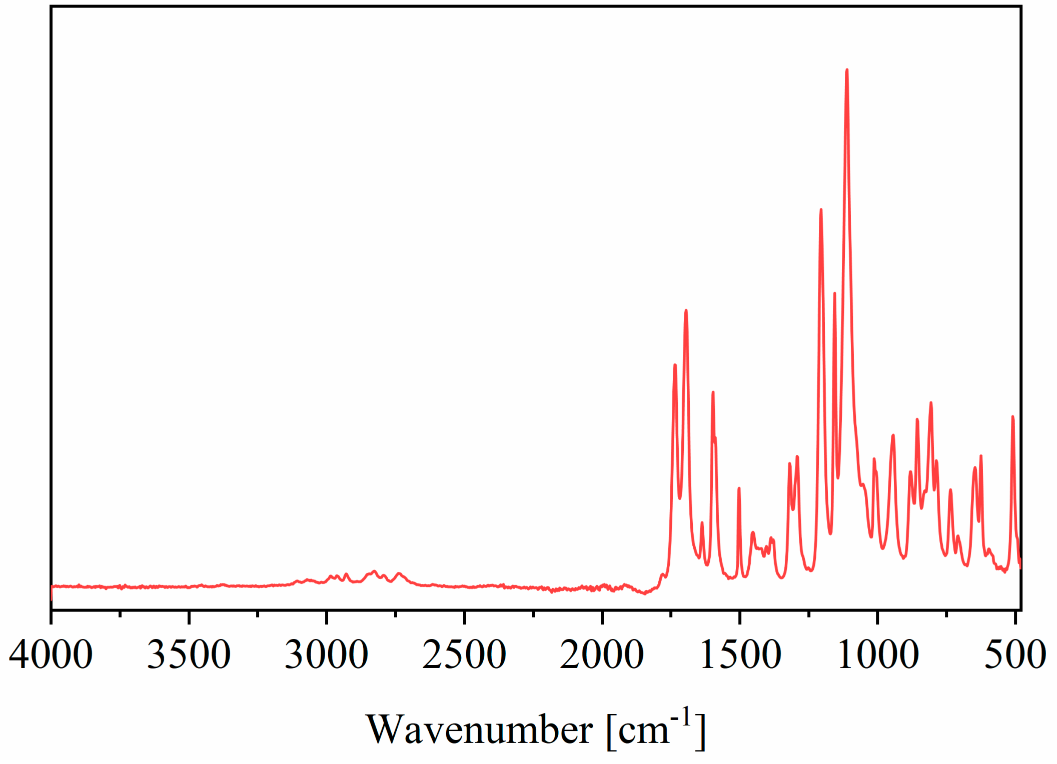


**Figure S1**: FT-IR spectrum of 4-FPMA

2. Imination of 4-formylphenyl methacrylate (4-FPMA) with JED (M-JED)

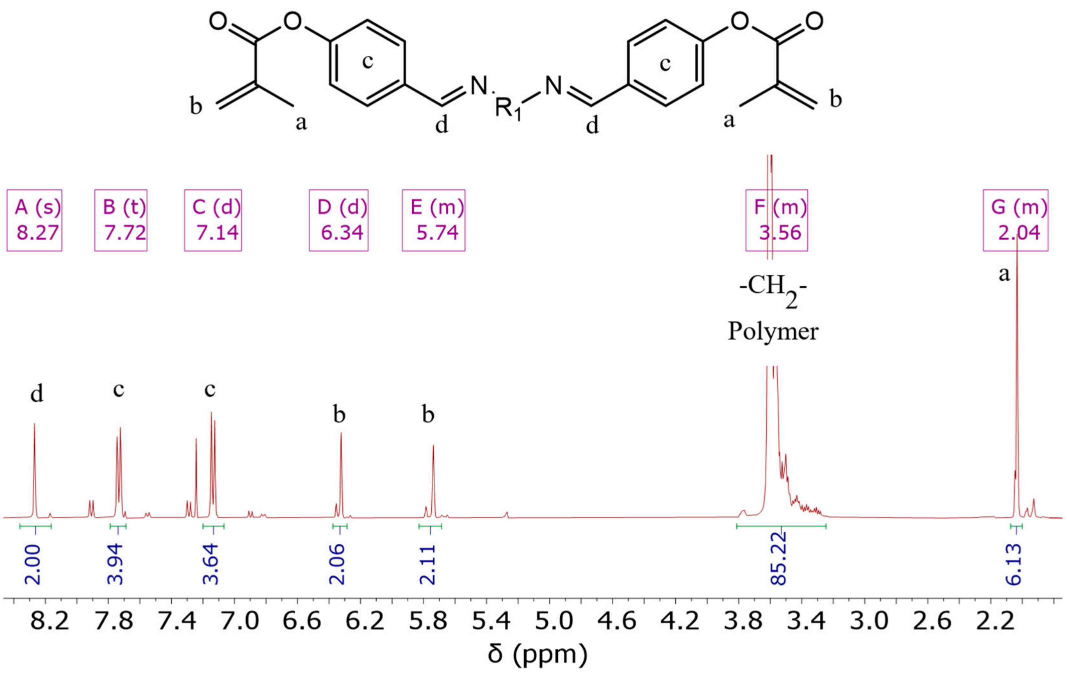


**Figure S2**: $^{1}$H-NMR spectrum of M-JED.

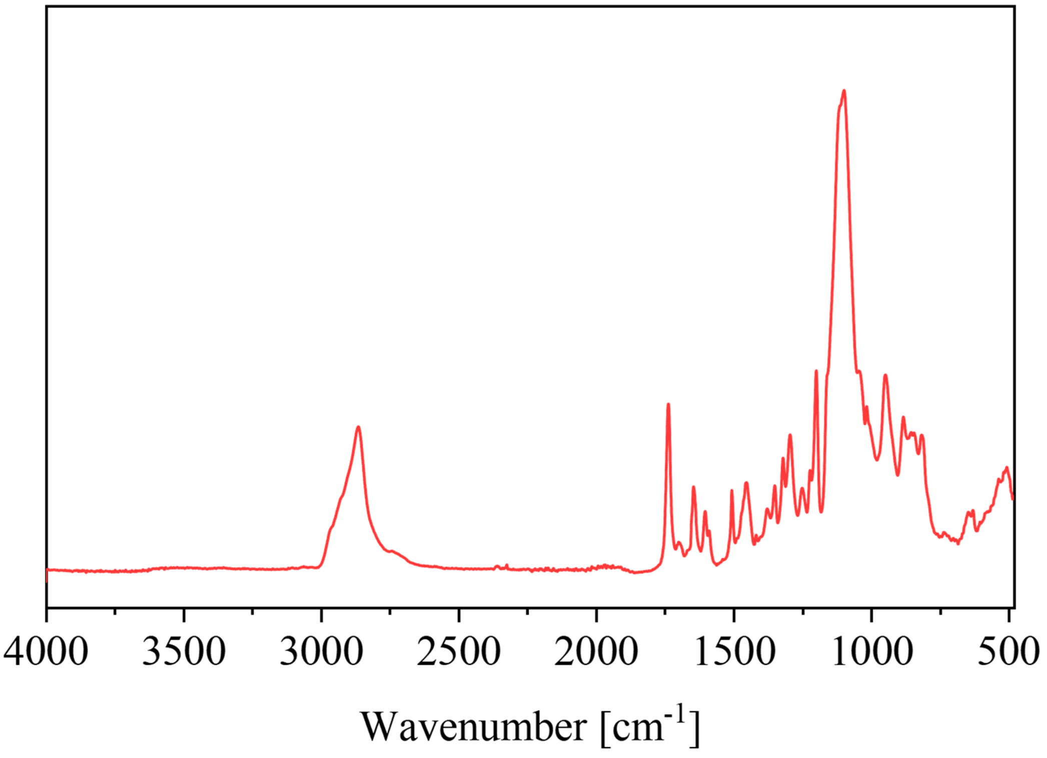


**Figure S3**: FT-IR spectrum of M-JED.

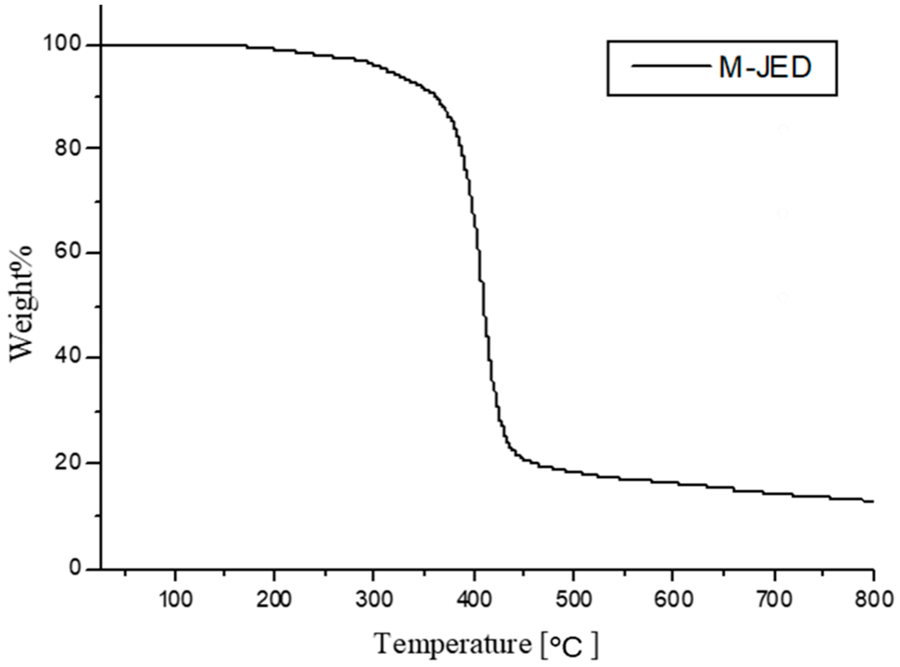


**Figure S4**: TGA thermogram of M-JED.

3. Synthesis of TOTDD-diol

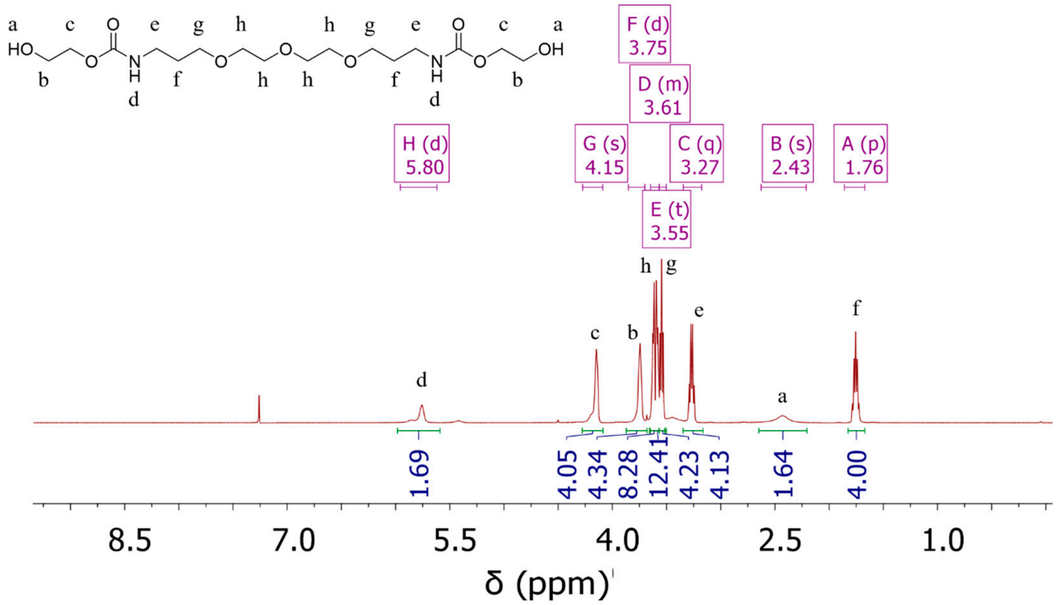


**Figure S5**: $^{1}$H-NMR spectrum of TOTDD-diol.

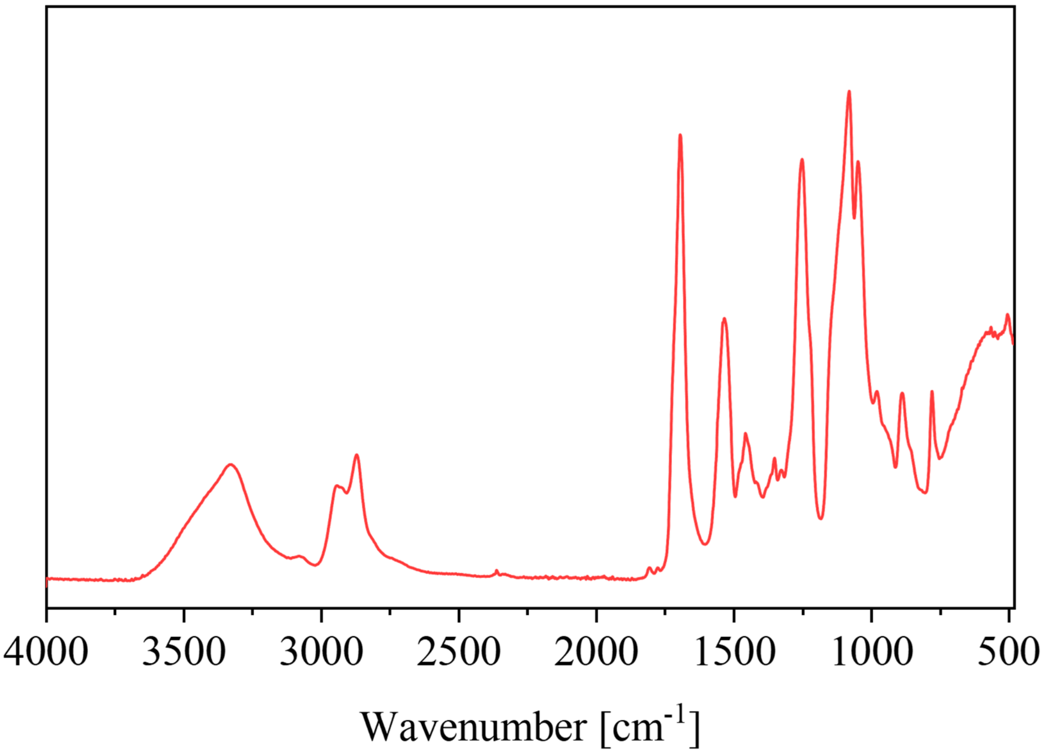


**Figure S6**: FT-IR spectrum of TOTDD-diol.

4. Synthesis of M-TOTDD

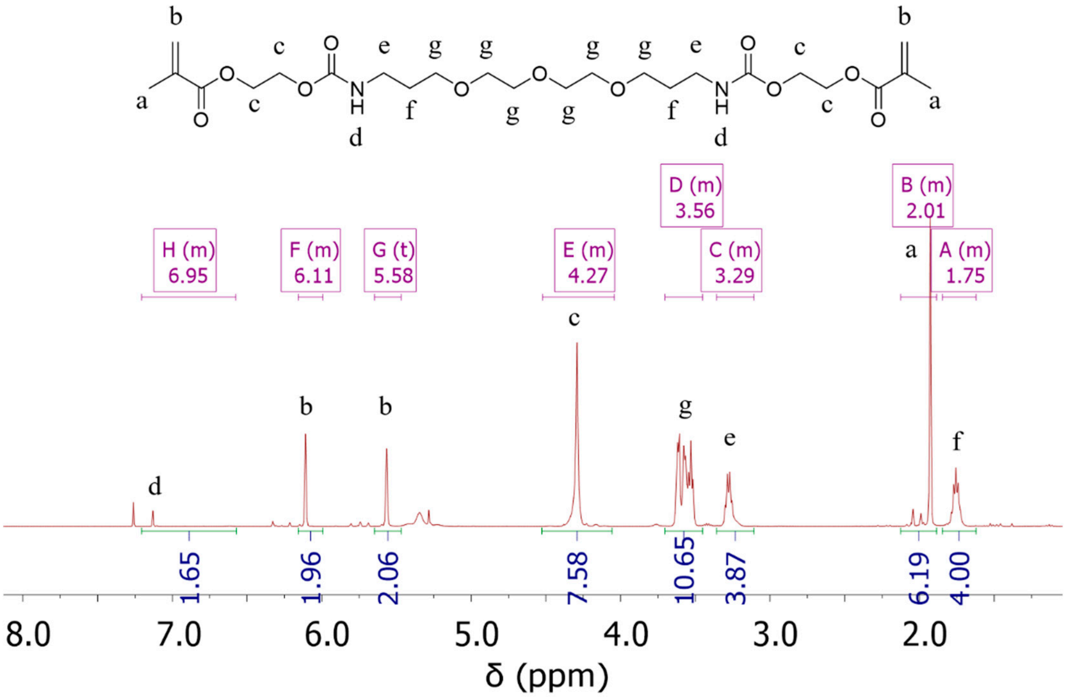


**Figure S7**: $^{1}$H-NMR spectrum of M-TOTDD.

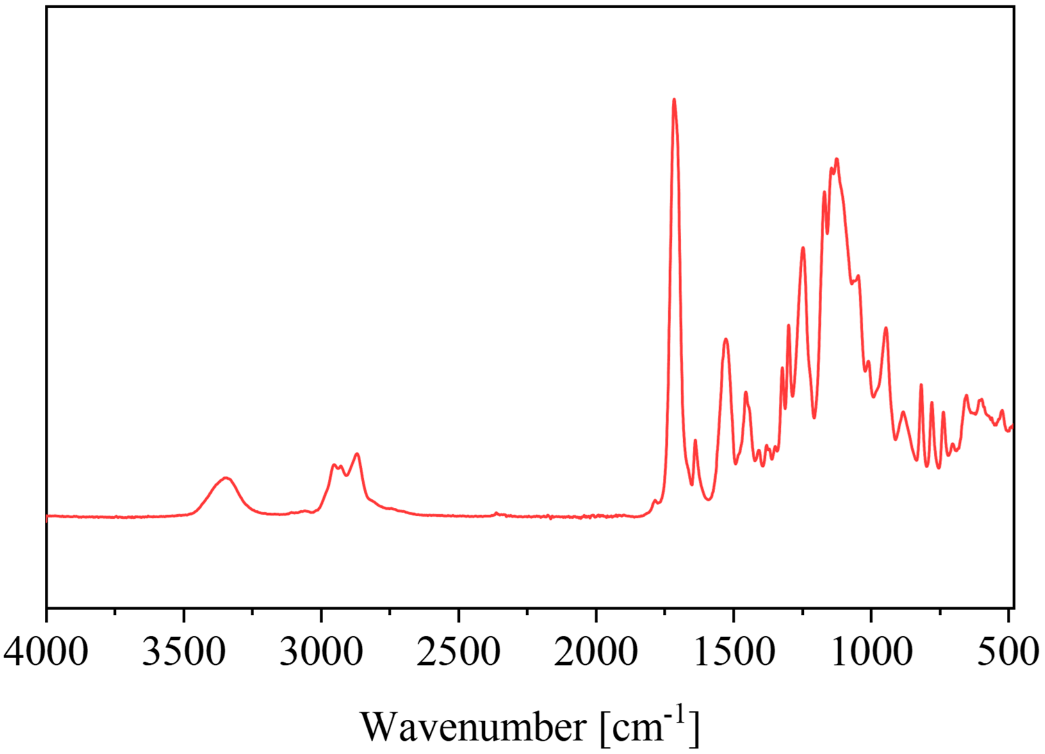


**Figure S8**: FT-IR spectrum of M-TOTDD.

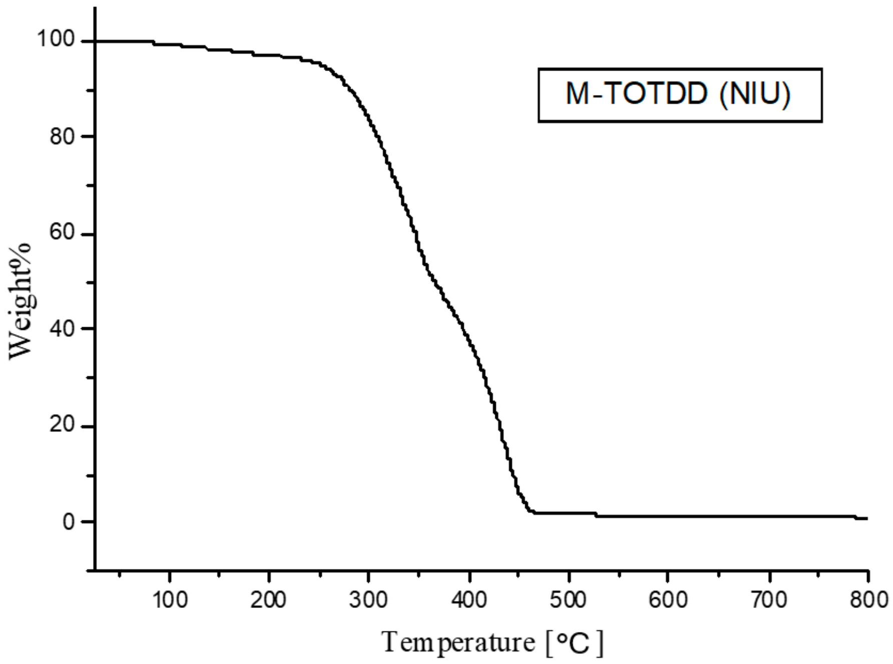


**Figure S9**: TGA thermogram of M-TOTDD.

5. FT-IR and TGA studies

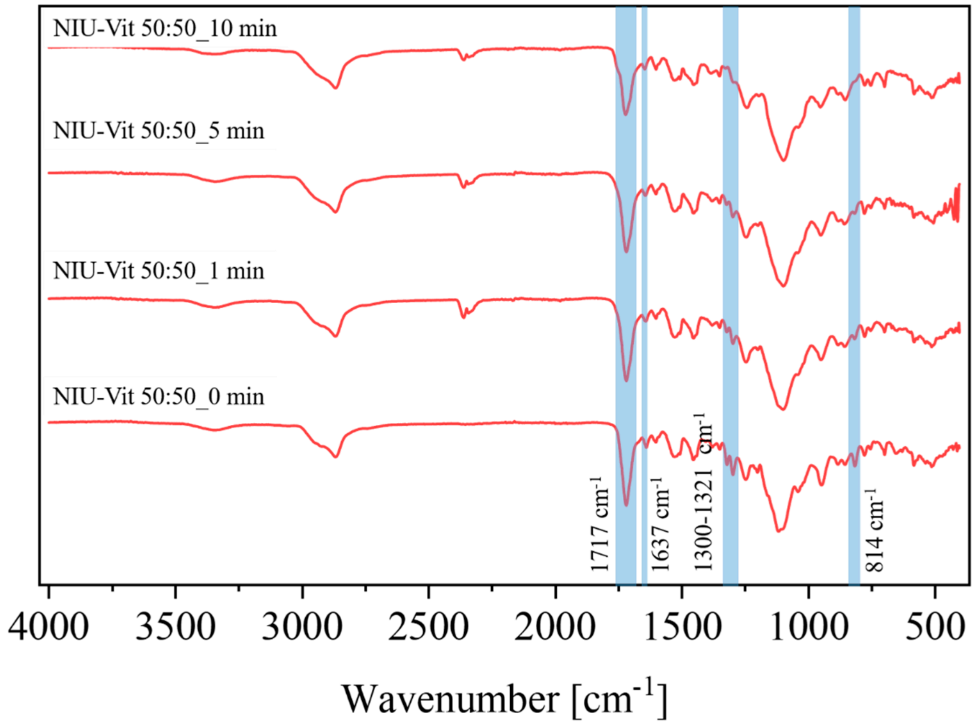


**Figure S10**: Full spectra of the effect of UV exposure time on photopolymerisation curing in NIU-Vit 50:50.

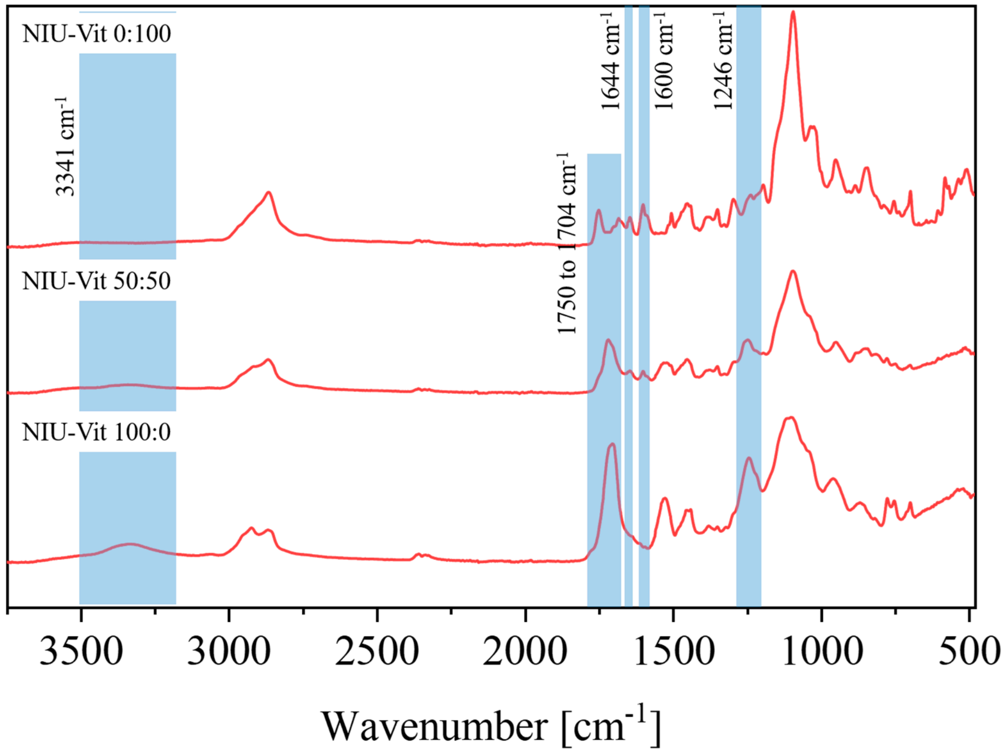


**Figure S11**: FT-IR spectra of the NIU-Vit networks with varying compositions.

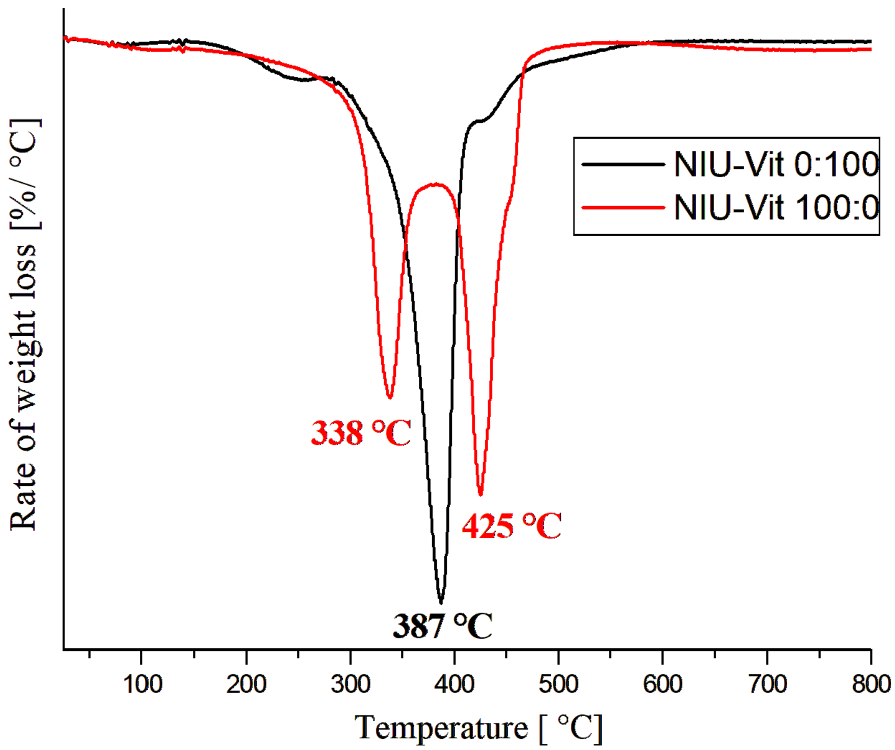


**Figure S12**: The DTG curve of NIU-Vit 100:0 and 0:100.

6. Determination of Gel Content

**Table S1**: Gel content of different NIU-Vit systems with cyclohexane:DCM 70:30 (v/v%).

| Entry No. | Sample Ratio | Gel Content [%] |
|---|---|---|
| 1 | NIU-Vit 30:70 | 90 |
| 2 | NIU-Vit 50:50 | 93 |
| 3 | NIU-Vit 70:30 | 96 |
| 4 | NIU-Vit 100:0 | 97 |

**Table S2**: Gel content of NIU-Vit 70:30 in different solvents.

| Entry No. | Solvent | Gel Content [%] |
|---|---|---|
| 1 | DCM | 93 |
| 2 | Cyclohexane | 99 |
| 3 | THF | 99 |
| 4 | Acetone | 97 |

7. Assessing thermal properties

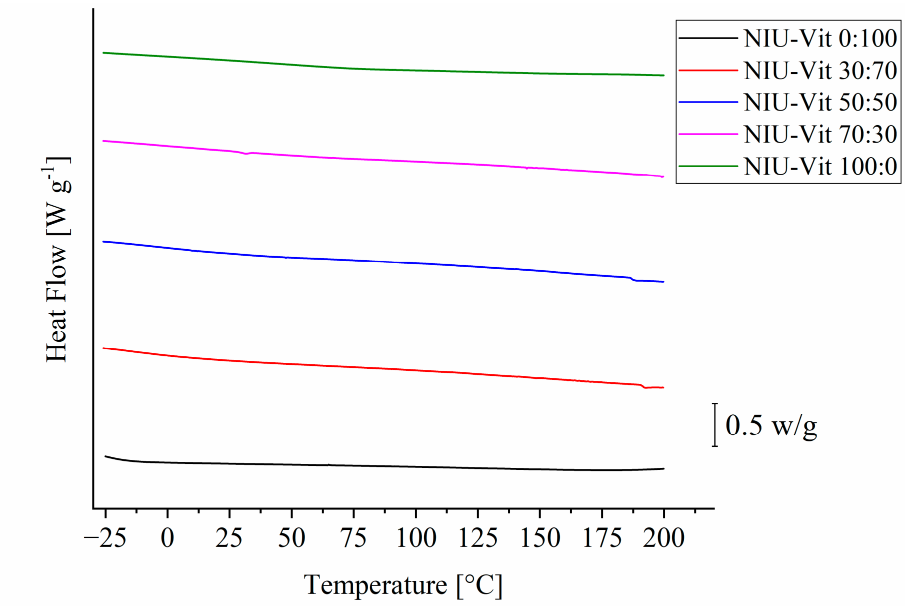


**Figure S13**: DSC thermograms of different NIU-Vit systems

**Table S3**: Characteristic degradation temperatures at 5, 50, 80 and 90 wt% loss, as well as the char residue of different NIU-Vit systems.

| Entry No. | Sample Name | $T_{5\%}$ ( °C) | $T_{50\%}$ ( °C) | $T_{80\%}$ ( °C) | $T_{90\%}$ ( °C) | Char Residue (wt% at 800 °C) |
|---|---|---|---|---|---|---|
| 1 | NIU-Vit 100:0 | 231 | 394 | 441 | 552 | 5.0 |
| 2 | NIU-Vit 70:30 | 304 | 413 | 440 | 455 | 5.1 |
| 3 | NIU-Vit 50:50 | 191 | 412 | 460 | - | 13.3 |
| 4 | NIU-Vit 30:70 | 283 | 415 | 560 | - | 17.8 |
| 5 | NIU-Vit 0:100 | 243 | 387 | 675 | - | 19.1 |

8. Uniaxial tensile testing

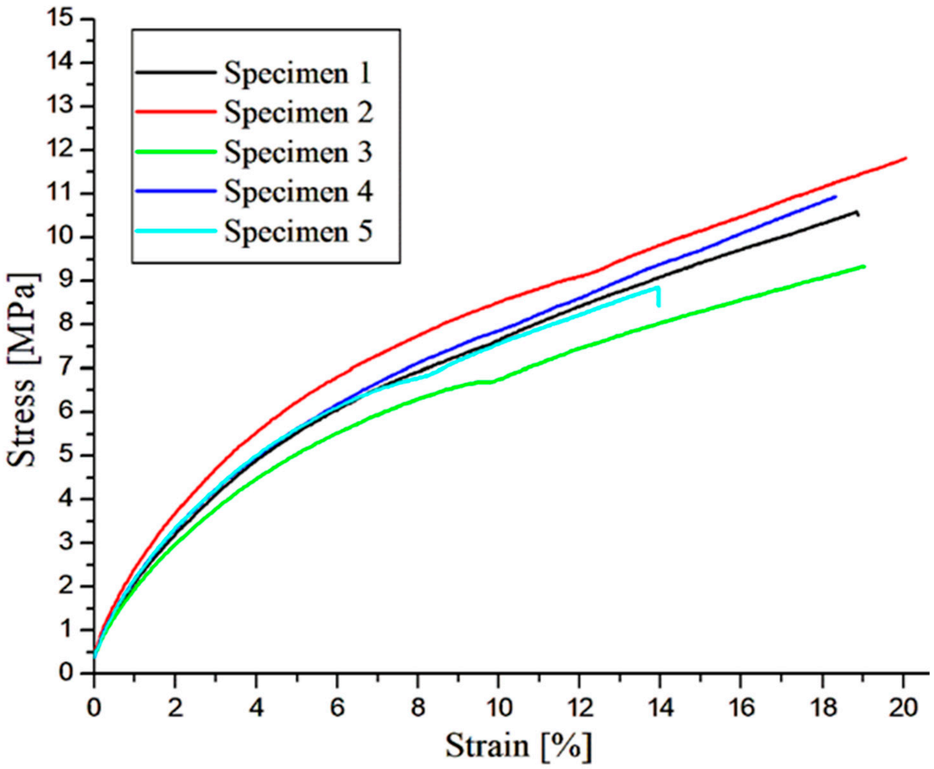


**Figure S14**: The stress-strain curves of the uniaxial tensile testing of five specimens of NIU-Vit 70:30.

**Table S4**: Results of tensile modulus after uniaxial tensile testing.

| Entry No. | Tensile Modulus [MPa] | Average E [MPa] | Std. Deviation [MPa] |
|---|---|---|---|
| 1 | 84.59 | 74.36 | 9.06 |
| 2 | 82.25 | | |
| 3 | 68.58 | | |
| 4 | 63.09 | | |
| 5 | 73.29 | | |

**Table S5**: Results of different tensile properties after uniaxial tensile testing.

| Entry No. | Stress at maximum force ($\sigma_m$) [MPa] | Strain at maximum force ($\varepsilon_m$) [%] | Stress at break ($\sigma_b$) [MPa] | Strain at break ($\varepsilon_b$) [%] |
|---|---|---|---|---|
| 1 | 10.6 | 18.85 | 10.5 | 18.88 |
| 2 | 11.8 | 20.06 | 11.8 | 20.06 |
| 3 | 9.3 | 19.03 | 9.3 | 19.03 |
| 4 | 10.9 | 18.33 | 10.9 | 18.33 |
| 5 | 8.8 | 13.95 | 8.4 | 13.98 |
| Average | 10.3 | 18.04 | 10.2 | 18.06 |

| | | | | |
|---|---|---|---|---|
| Std. Deviation | 1.2 | 2.38 | 1.3 | 2.36 |

9. Self-healing assessment test

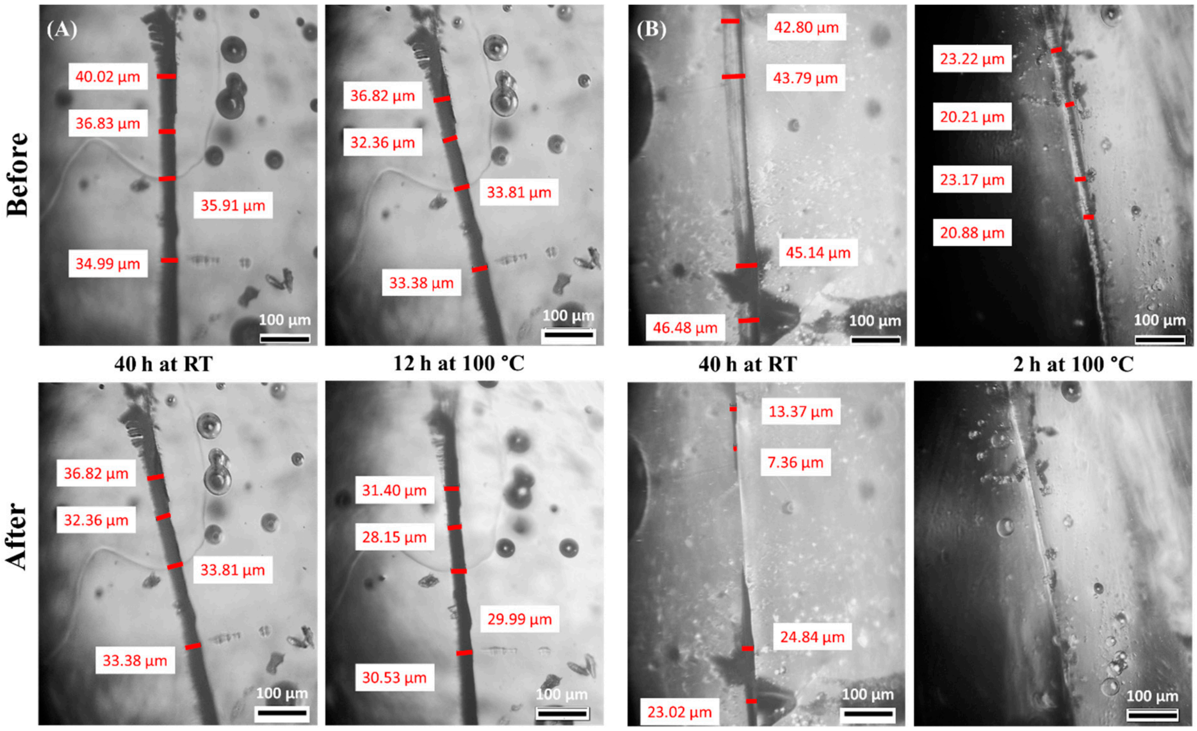


**Figure S15**: Control samples for surface self-healing behavior consisting of before and after microscopy images of (A) NIU-Vit 100:0 and (B) NIU-Vit 0:100

**Table S6**: Results of surface self-healing behavior of NIU-Vit 70:30 with controls of NIU-Vit 0:100 and 100:0 at room temperature (23 ± 2 °C und 50 ± 10 % r.h).

| Entry Nr. | Sample | Average Scratch Width [µm] | | | Difference [µm] | Change [%] |
|---|---|---|---|---|---|---|
| | | 0 h | 24 h | 40 h | | |
| 1 | NIU-Vit 100:0 | 36.93 | 34.64 | 34.09 | - 2.84 | -7.7 |
| 2 | NIU-Vit 70:30 | 30.37 | 8.77 | - | - 30.37 | -100.0 |
| 3 | NIU-Vit 0:100 | 44.55 | - | 17.15 | - 27.40 | -61.5 |

**Table S7**: Results of surface self-healing behavior of NIU-Vit 70:30 with controls of NIU-Vit 0:100 and 100:0 at 100 °C.

| Entry Nr. | Sample | Average Scratch Width [µm] | | | | Difference [µm] | Change [%] |
|---|---|---|---|---|---|---|---|
| | | 0 h | 2 h | 6 h | 12 h | | |
| 1 | NIU-Vit 100:0 | 34.09 | 33.01 | 31.18 | 30.02 | - 4.07 | -11.9 |
| 2 | NIU-Vit 70:30 | 41.54 | - | - | - | - 41.54 | -100.0 |
| 3 | NIU-Vit 0:100 | 21.87 | - | - | - | - 21.87 | -100.0 |

10. Prospects for recyclability of the NIU-Vit systems

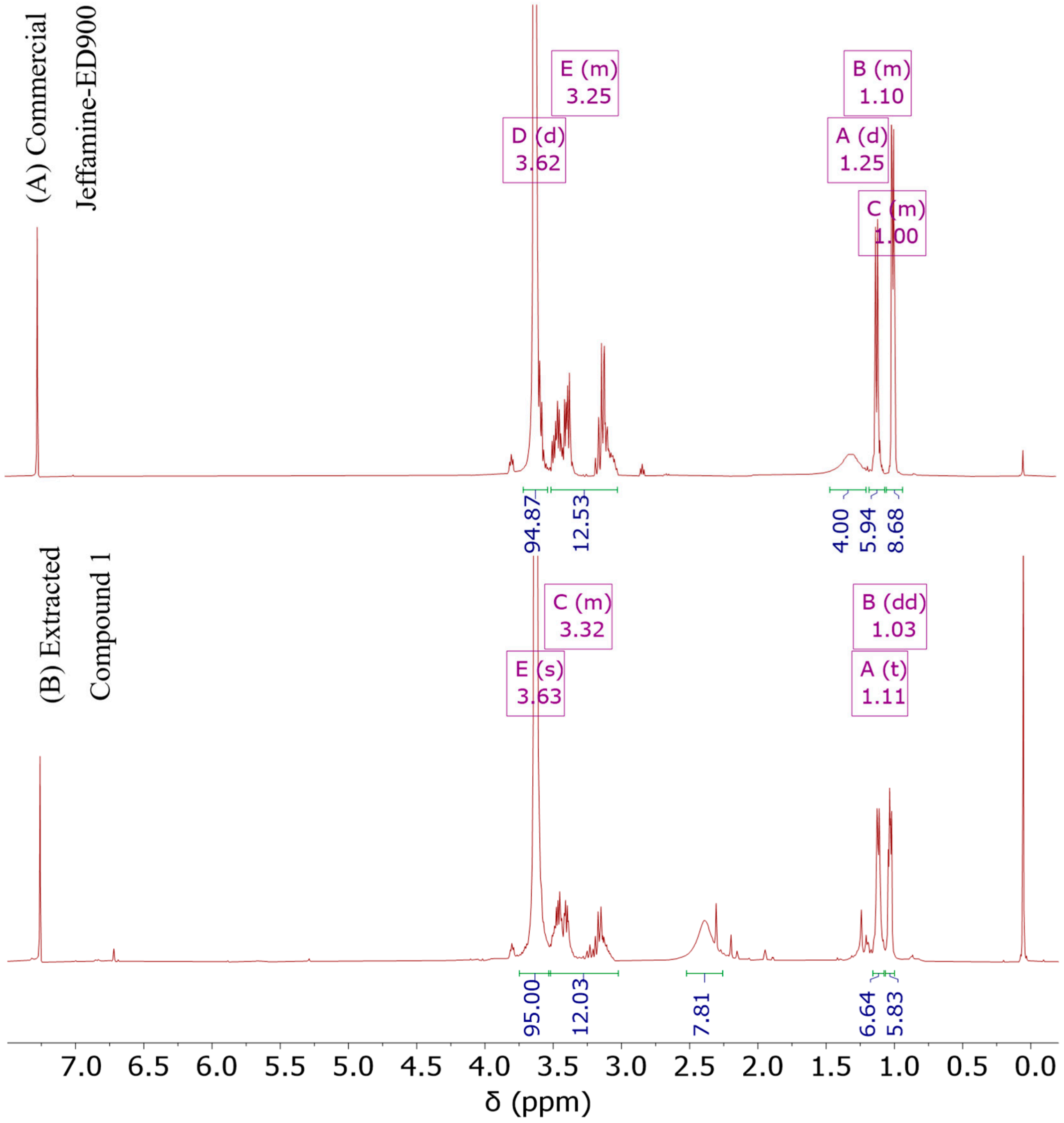


**Figure S16**: The NMR comparison of (A) commercial Jeffamine ED-900 and (B) Extracted Compound 1 (EC1).

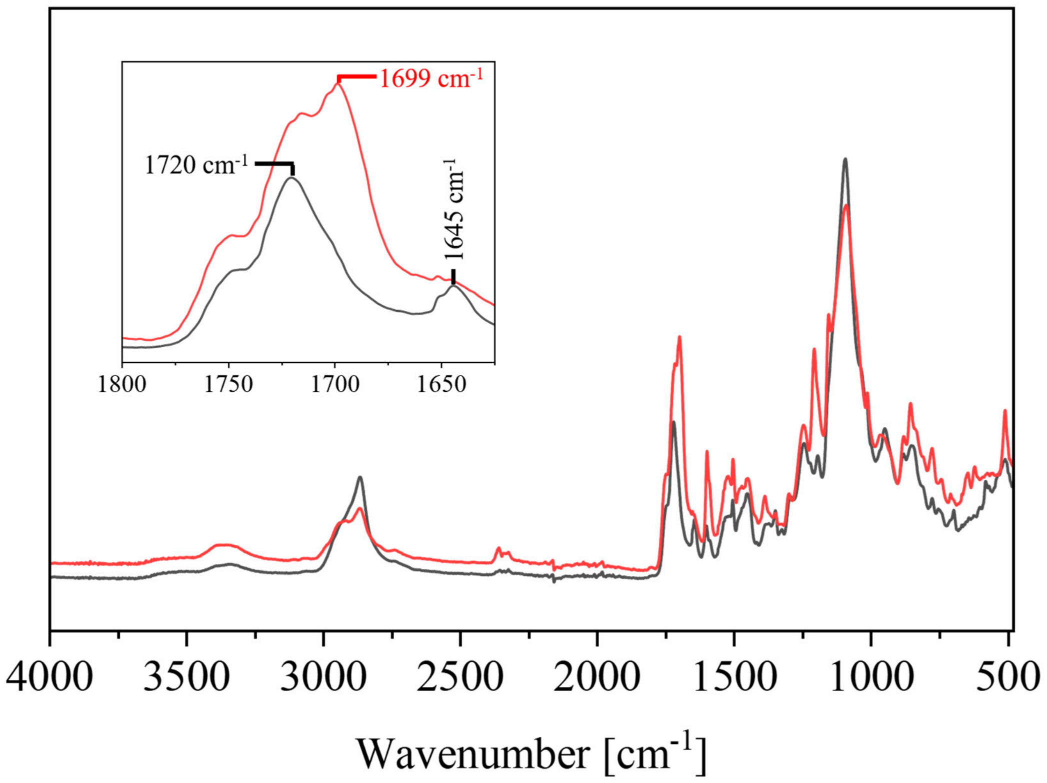


**Figure S17**: FT-IR comparison of NIU-Vit 25:75 before (in black) and after (in red, SF1) acidic hydrolysis.

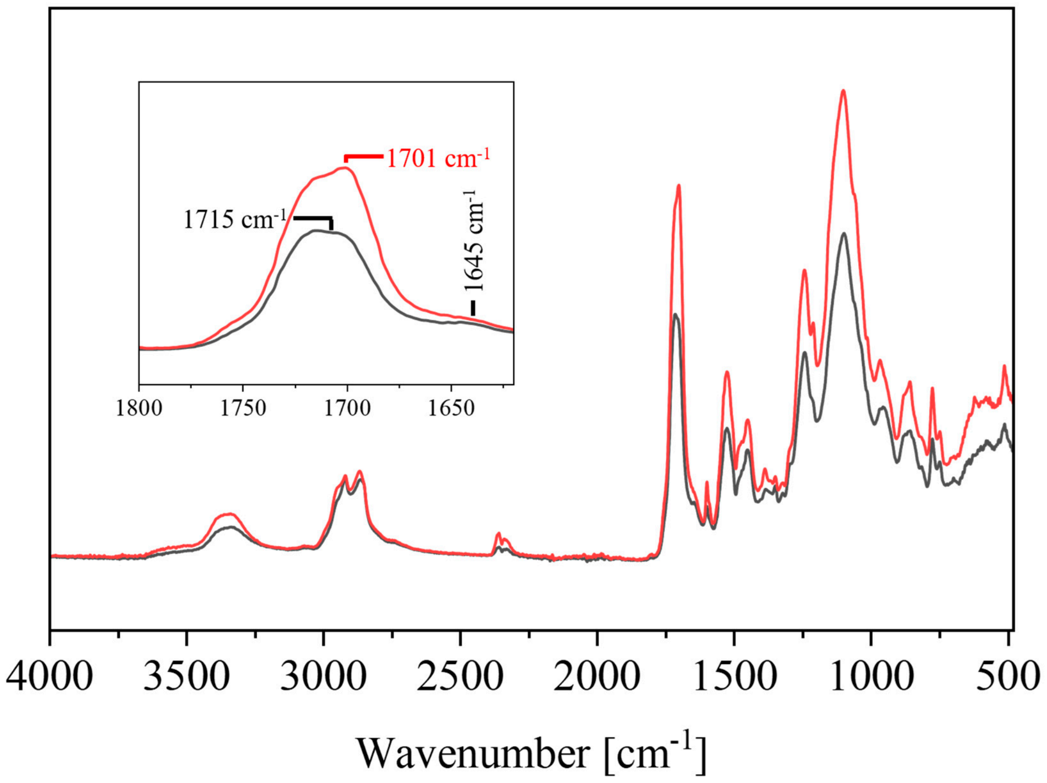


**Figure S18**: FT-IR comparison of NIU-Vit 75:25 before (in black) and after (in red, SF2) acidic hydrolysis.

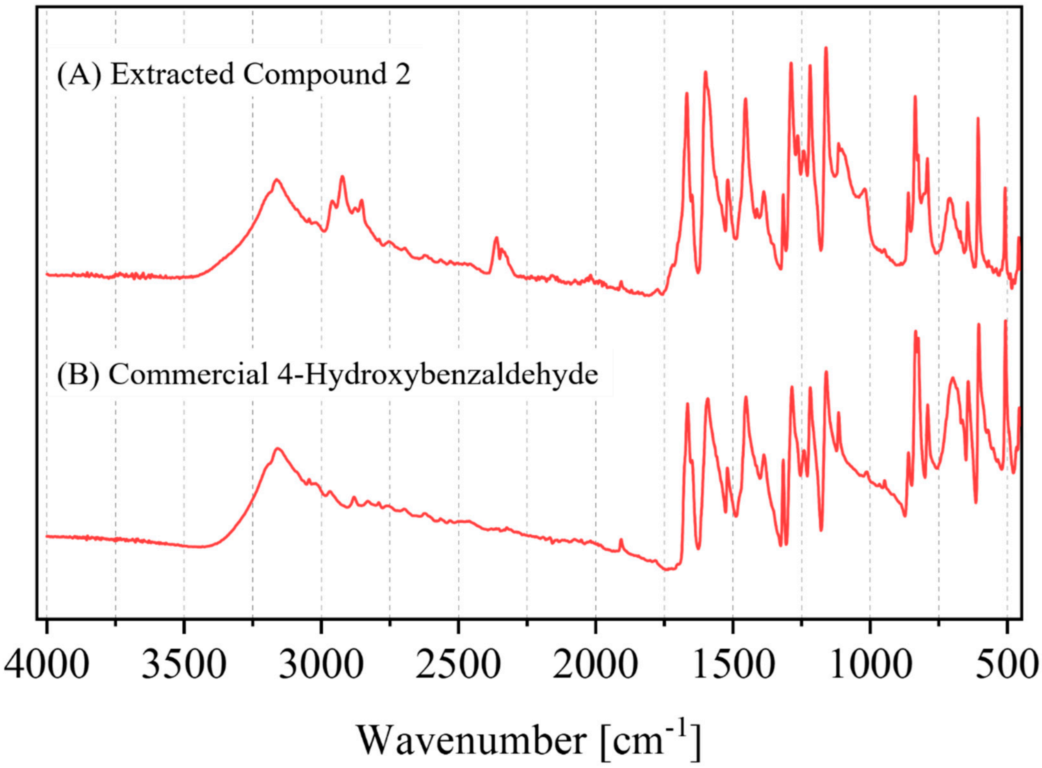


**Figure S19**: FT-IR comparison between (A) Extracted Compound 2 (EC2) at neutral pH and (B) commercial 4-HBA as reference

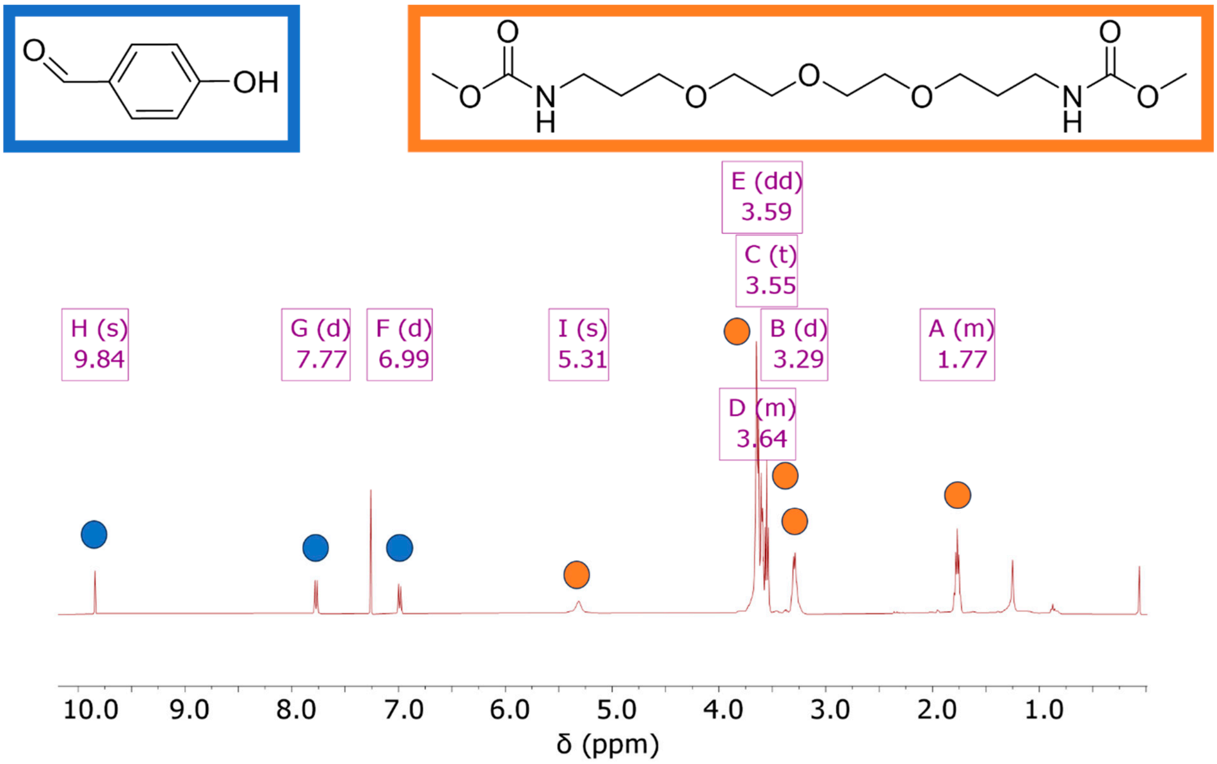


**Figure S20**: $^{1}$H-NMR of the Extracted Compound 3 (EC3) at acidic pH after basic alcoholysis.

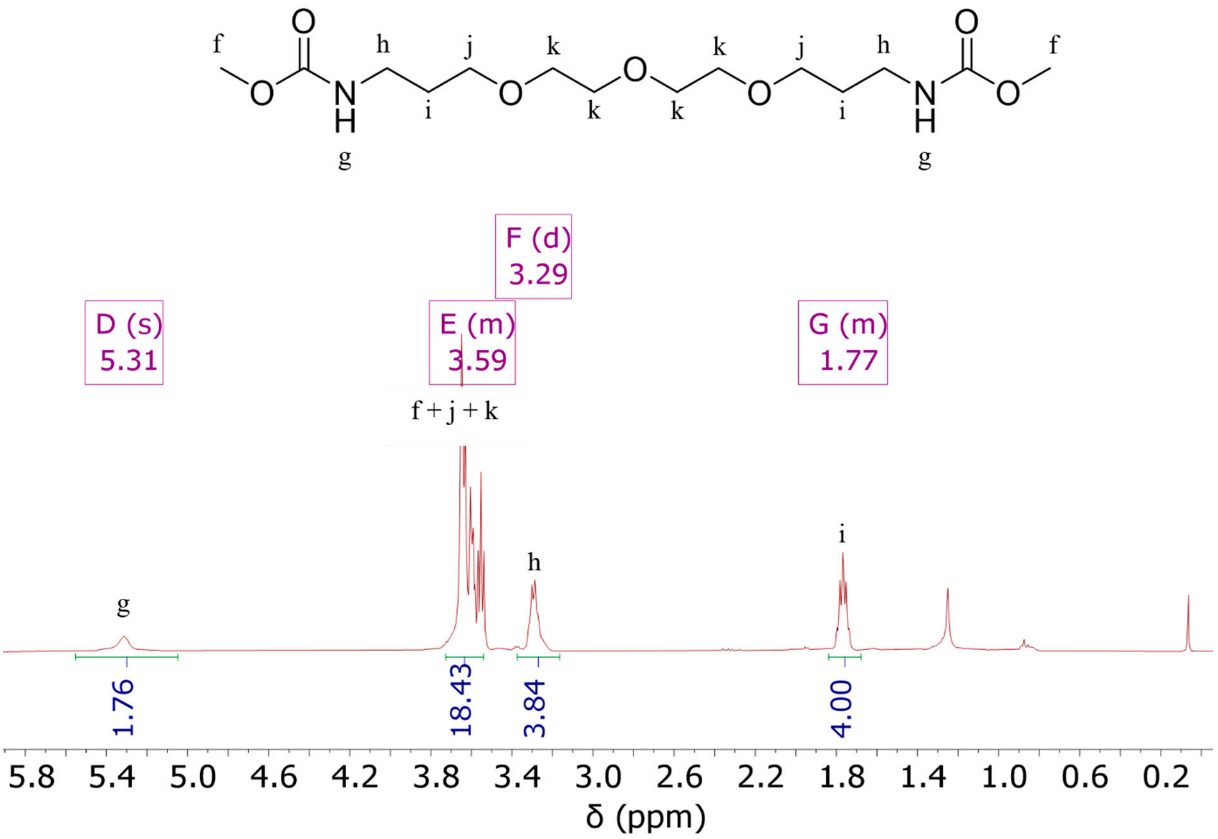


**Figure S21**: $^{1}$H-NMR of the extracted of bis(methyl carbamate) of TOTDD after basic alcoholysis.

11. Shape memory performance test

**Table S8**: The parameters and results for the bending test with a custom technique to quantify the shape memory performance of NIU-Vit 70:30 before and after permanent reprogramming.

Before Permanent Programming

| Specimen No. | $\theta_f$ | $\theta_r$ | Shape fixity ratio ($R_f$) | Shape recovery ratio ($R_r$) |
|---|---|---|---|---|
| 1 | 165.5 | 3.0 | 91.9 | 98.3 |
| 2 | 169.7 | 1.6 | 94.3 | 99.1 |
| 3 | 168.7 | 4.1 | 93.7 | 97.7 |
| Mean | | | 93.3 | 98.4 |
| Std. Deviation | | | 1.3 | 0.9 |

After Permanent Programming

| Specimen No. | $\theta_f$ | $\theta_r$ | $\theta_0$ | Shape fixity ratio ($R_f$) | Shape recovery ratio ($R_r$) |
|---|---|---|---|---|---|
| 1 | 174.1 | 150.2 | 148.9 | 96.7 | 95.7 |
| 2 | 177.5 | 156.3 | 154.5 | 98.6 | 92.7 |
| 3 | 176.5 | 153.4 | 146.2 | 98.1 | 78.8 |
| Mean | | | | 97.8 | 89.1 |
| Std. Deviation | | | | 0.6 | 3.3 |

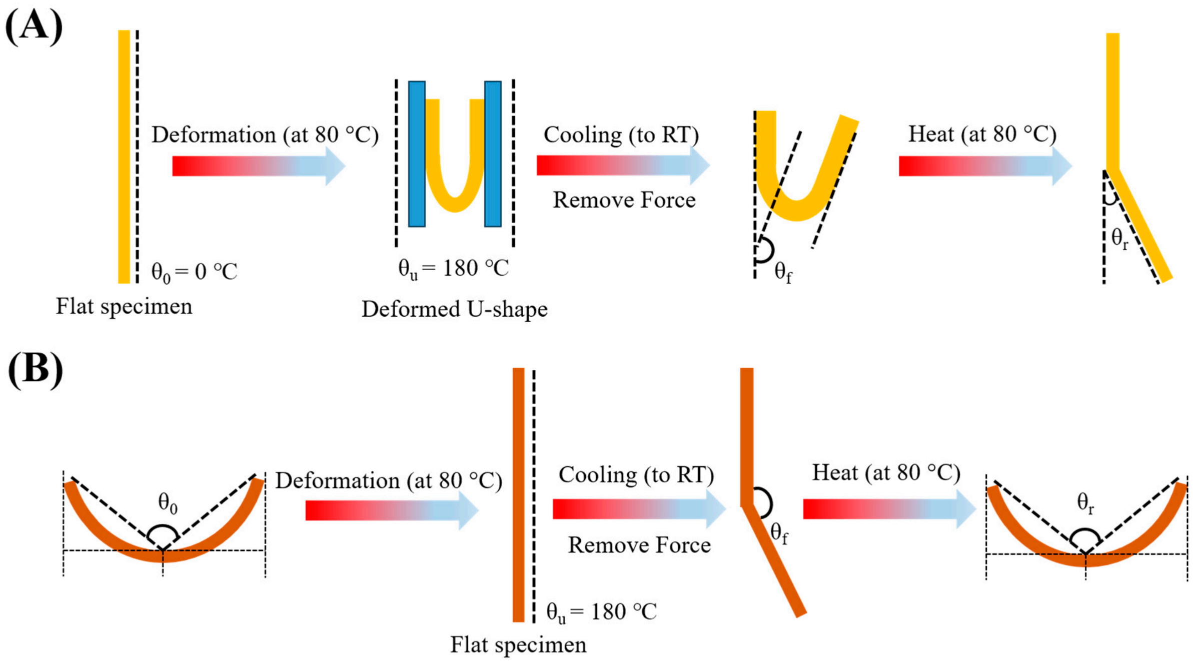


**Figure S22**: The bending test with a custom technique was used to quantify the shape memory performance of NIU-Vit 70:30 A) before and B) after permanent reprogramming

**Video S1**: Shape recovery to the original permanent shape (flat) from a temporary curved shape (**Figure 4A2-3**). Video speed has been increased by ~5x.

**Video S2**: Shape recovery to the original permanent shape (curve) from a temporary flat shape (**Figure 4A5-6**). Video speed has been increased by ~4x.
